# Chemical Valorization of Textile Waste: Advancing Sustainable Recycling for a Circular Economy

**DOI:** 10.1021/acsomega.4c10616

**Published:** 2025-03-19

**Authors:** Joyjit Ghosh, Md. Reazuddin Repon, Nishat Sarmin Rupanty, Tanvir Rahman Asif, Mohammed Islam Tamjid, Vladimir Reukov

**Affiliations:** † Department of Textiles, Merchandising and Interiors, University of Georgia, Athens, Georgia 30602, United States; ‡ Department of Bioproducts and Biosystems, School of Chemical Engineering, Aalto University, Vuorimiehentie 1, Espoo 02150, Finland; § Department of Textile Engineering, Ahsanullah University of Science and Technology, 141 & 142, Love Road, Tejgaon Industrial Area, Dhaka 1208, Bangladesh; ∥ Department of Textile and Apparel Management, University of Missouri, Columbia, Missouri 65211, United States

## Abstract

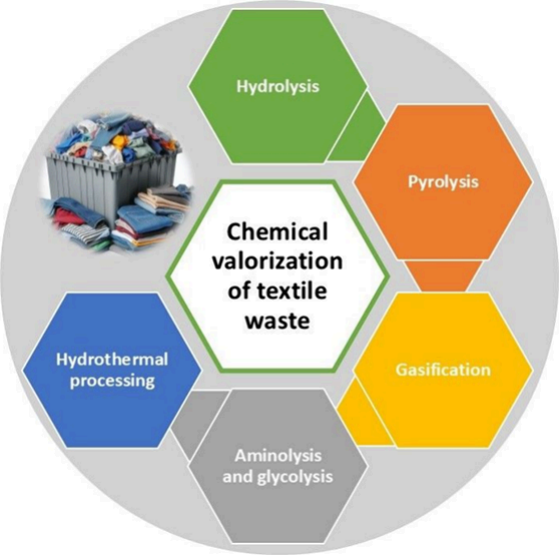

As textile production continues to grow worldwide, managing the mounting waste generated by this industry is becoming an urgent environmental concern. Globally, over 92 million tons of textile waste are produced annually, much of which is incinerated or disposed of in landfills, contributing to greenhouse gas emissions, soil and water contamination, and ecosystem harm. This review explores how chemical and biotechnological methods, such as acid hydrolysis (achieving up to 70% glucose recovery) and enzymatic recycling (reducing energy consumption by approximately 20% compared to conventional methods), can transform textile waste into valuable resources, fostering a shift toward a circular economy that minimizes reliance on virgin materials. However, the diverse nature of textile waste—particularly in mixed fibers and materials treated with various finishes and additives—adds complexity to recycling processes, often necessitating specific pretreatment steps to ensure both efficiency and economic viability. Scalable solutions such as advanced solvent recovery systems, optimized pretreatment techniques, and fluidized-bed pyrolysis (which can increase bio-oil yields by up to 25% compared to fixed-bed reactors) play crucial roles in making textile recycling more sustainable and adaptable at an industrial scale. By addressing these technical and financial challenges, the industry can improve the efficiency and sustainability of textile recycling practices, reducing waste and contributing to environmental resilience. This review also suggests several future directions to enhance scalability and compatibility with environmental goals, highlighting the potential for these technologies to create valuable secondary materials and support greener practices in textile waste management. Through continued innovation and a commitment to sustainable practices, the textile industry can better balance resource recovery with economic feasibility, unlocking substantial opportunities to mitigate environmental impact and support a more resource-efficient, sustainable future.

## 1 Introduction

The manufacturing of textiles has surged due to the expanding worldwide population, leading to a substantial amount of waste. The fashion industry generates annual global clothing waste valued at $400 billion.^1^ In the United States, textile waste reached 11.9 million tons in 2007, making up 4.7 wt % of municipal solid waste (MSW), with a recovery rate of only 15.9%. The overall MSW distribution showed 54% went to landfills, 13% was incinerated, and 33% was either recycled or composted.^2^ Across the globe, textile waste constitutes between 1.0–5.1% of MSW, with varying percentages by region: 7% in Kaikoura, New Zealand, 4.95% in the U.S., 1.3% in Beijing, China, and 4.7% in Bhutan, inclusive of leather materials.^3^ The cumulative impact amounts to more than 184 billion pounds of textile waste produced globally each year.^4^ The conversion of textile waste into value-added products is necessary to address this issue as it can aid in the shift to a circular economy and minimize pollution.^5^ Particularly when it comes to burning and landfilling, which contaminate soil and water and produce greenhouse gases, textile waste has a substantial negative influence on the environment. These actions exacerbate environmental deterioration and global warming.^6^ Discarded clothing and household goods made of biobased materials make up postconsumer biobased textile waste. The best way to manage this kind of trash is through organic recycling, however, because multimaterial textiles are still present, selective collection and sorting still present difficulties. Recent advancements in textile waste recycling such as cotton focus on three primary methods: mechanical, chemical, and biological recycling. Mechanical recycling involves processes like opening, spinning, and weaving to physically decompose waste cotton without altering its chemical structure, allowing it to be repurposed into fibers, yarns, fabrics, or nonwovens, with applications in blended yarns and composite reinforcements.^7^ Chemical recycling uses reagents to degrade or depolymerize polymers into smaller molecules or reactive monomers, which can then be polymerized into new materials. This process emphasizes cellulose extraction, given cotton’s high cellulose content (up to 95%).^8^ Techniques include nonderivative methods, which break hydrogen bonds in cellulose, and derivative methods, which chemically modify cellulose to produce advanced materials like adsorptive products and flexible electronics. Biological recycling employs physical, biochemical, or thermochemical methods to convert waste cotton into biofuels such as bioethanol and biogas, which are valued for their renewability, biodegradability, and cleaner exhaust emissions.^9^ These methods represent promising approaches to utilizing waste cotton as a renewable energy source to reduce reliance on fossil fuels. These strategies align with emerging trends in sustainable resource utilization, emphasizing high-value applications and the transition to a circular economy.^10^ In order to maximize recycling efficiency and reduce environmental effects, methods such as acid hydrolysis and enzymatic processes are investigated for regaining important components from textile waste. By encouraging sustainable medical textiles through natural, environmentally friendly substitutes, the addition of coriander extract to Cotton bandages improves their antibacterial and wound-healing qualities and supports the valorization of textile waste.^11^ Biodegradable and compostable materials are crucial for this process.^12^ Inconsistent regulations on waste management provide substantial issues for the textile industry. Diverse rules and techniques are implemented by different nations to manage textile waste, which results in inefficiencies and gaps in compliance. This lack of consistency can make it more difficult to create frameworks for efficient waste management, which will impede attempts to encourage sustainability and ethical behavior throughout the world’s textile supply chain. By converting industrial waste into reusable resources and encouraging eco-friendly manufacturing at the worker level, the garment industry may address environmental concerns by implementing sustainable methods like textile waste valorization.^13^ The operations of sorting and recycling are made more difficult by the varied content of textile waste. Variations in finishes, dyes, and fabric kinds can impact how well-recycled materials work. For recyclers hoping to turn textile waste into high-quality secondary raw materials, this discrepancy presents difficulties.^14^ While cutting-edge technologies like thermal, biological, and chemical recycling provide promising approaches to managing textile waste, their implementation frequently runs into problems with scalability and high costs. Precise control in chemical valorization processes is essential for optimizing material recovery and attaining reliable recycling results, as spindle speed optimization improves yarn quality.^15^ Many of these technologies demand substantial infrastructure and expenditure, which may not be possible for many areas or firms, especially smaller ones.^16^ By enhancing yarn quality and uniformity, increasing the hardness of roller cots in textile spinning could lower material waste and promote textile waste valorization through effective manufacturing methods.^17^

Global garbage generation has increased dramatically as a result of urbanization, economic expansion, and population growth. The World Bank projects a 70% increase in waste generation by 2050, which poses major dangers to the environment and human health. Adding value to waste materials while reducing negative effects is waste valorization, which has emerged as a viable approach to move toward a circular economy. Food, agricultural, textile, plastics, and electronics waste streams are the main ones that can be analyzed to reveal current valorization technologies, adoption hurdles, and workable ideas for improving sustainability.^18^ Significant waste is produced by the textile industry, which is usually disposed of using methods that harm the environment, such as incineration and landfilling. In order to reduce pollution and promote sustainability, this trash must be transformed into goods with added value. Diverse classifications and origins of textile waste present opportunities for recycling and value addition in industries such as energy production, composites, textiles, and packaging, all while addressing the difficulties associated with these activities.^19^ Natural fiber-reinforced composites are becoming more and more valued for their high rigidity, low weight, and affordability, which make them appropriate for use in building and transportation. The valorization of materials like flax, hemp, and jute fabrics is encouraging the development of innovative processing technologies to create value-added goods, while also promoting sustainability by repurposing waste in light of the growing amount of textile waste.^20^ Although water is necessary for life, the textile industry heavily uses it and contaminates aquatic environments. Industrial dye spills damage water quality by increasing COD and BOD levels, interfering with photosynthesis, endangering the food chain, and harming human health. Modern biotechnological developments have improved the efficacy of bioremediation, which uses the enzymatic action of different organisms to treat these toxins. Bioremediation is a sustainable approach. This method promotes sustainability and raises everyone’s quality of life while providing a competitive substitute for conventional physicochemical treatments.^21^

The problems of mixed textile waste are successfully addressed by chemical recycling of polyester textiles by glycolysis over a ZnO catalyst with microwave assistance. With this method, polyester and Spandex can be quickly depolymerized into their monomers in as little as 15 min. Cotton and nylon can then be separated by solvent dissolution. In the textile sector, this approach supports a circular economy by encouraging sustainable recycling, minimizing environmental impact, and being economically feasible.^22^ The production of textile waste poses serious environmental problems, which must be addressed by upcycling cotton-derived textile waste into value-added chemicals via enzymatic hydrolysis. Efficient pretreatment techniques are essential because they improve cotton’s poor glucose conversion, which is impeded by the material’s high crystallinity and dye content. These techniques can enhance the sustainability of the enzymatic process by decreasing the requirement for large enzyme loads, energy consumption, and chemical inputs and increasing enzyme accessibility. All things considered, developing these methods is essential to converting textile waste into useful biobased building blocks, promoting a circular economy, and reducing environmental impact.^23^ Reducing water and chemical consumption, minimizing wastewater pollution, and turning textile waste into valuable materials are all made possible by the sustainable cationic dyeing method for cotton fabric, which is essential for chemical waste valorization and falls under the circular economy paradigm.^24^ Using cotton and PET textiles as a substrate in fungal solid-state fermentation to manufacture cellulase—A. niger CKB was chosen for this purpose due to its high cellulase activity—and autoclaving to improve enzyme accessibility to the fibers is a unique strategy for valorizing textile waste.^25^ Chemical processing can improve the characteristics of textile fibers. For example, the creation of textiles that are antibacterial and UV-protective shows how to effectively value textile waste by providing low-cost, commercially viable functional materials that significantly lower harmful UV exposure and fight bacterial strains. This method emphasizes how natural waste can be recycled to improve the sustainability of textile products.^26^ In order to develop sustainable adsorbent materials that efficiently remove contaminants from wastewater, address pressing environmental issues, and improve resource recovery, biowaste must be valued through the hydrothermal carbonization process.^27^ Thermochemical pyrolysis is an efficient method of converting heterogeneous textile waste into valuable products. It addresses the economic challenges associated with recycling while allowing material and energy recovery. However, more research and development is necessary to maximize the process’s potential as a sustainable waste management solution.^28^

This review provides a thorough overview of the most pertinent and efficient methods by focusing on analyzing the numerous chemical valorization procedures that are used to convert textile waste into useful commodities. It also explores the elements that have a major impact on the effectiveness and success of these processes, including the kind of catalysts used, the particular circumstances needed for the reactions, and the makeup of the waste material. In order to identify the practical viability of various chemical techniques, the review also evaluates their overall performance by assessing key criteria such as product output, energy consumption, and the sustainability of each process. Additionally, it draws attention to the difficulties encountered in scaling these methods for industrial application, notably with regard to operational complexity and economic viability. In order to maximize this chemical valorization processes’ efficiency, lessen their negative effects on the environment, and raise sustainability levels generally, the paper concludes by examining possible directions for future study. Table 1 highlights certain recent research in textile waste valorization.

**Table 1:** Summary of Recent Research in Textile Waste Valorization

Research focus	Key findings	Ref.
Statistics and effects of global textile waste	Landfills are overflowing with textile waste, which releases greenhouse gases and contributes to water and soil contamination due to its low recovery rates. The complicated nature of this material, which includes synthetic fibers and colors, makes recycling it more difficult and worse for the environment.	(1)
Recycling textile waste mechanically	In order to encourage sustainability and reduce waste, reusable textile waste is mechanically broken down into fibers and yarns. These are then used to make new threads, fabrics, or for industrial purposes.	(7)
Textile chemical recycling techniques	The breakdown of polymers into monomers, known as polymer depolymerization, highlights the extraction of cellulose from textiles, particularly those made of cotton. To promote sustainability and lessen textile waste, this extracted cellulose is recycled into high-value goods including innovative composites, biodegradable materials, and regenerated fibers.	(8)
Biological recycling approaches	Reusing textiles to make biofuels converts trash into biodegradable and renewable energy sources, like biogas and bioethanol. While supporting the circular economy, this method lessens reliance on fossil fuels, cuts greenhouse gas emissions, and offers a sustainable way to manage textile waste.	(9)
Natural fiber-reinforced composites	High-rigidity, lightweight composites with strength, durability, and environmental benefits are used in the construction and automotive industries. They are frequently constructed from natural fibers like hemp or flax.	(29)
Bioremediation for textile dye pollution	Enzymatic treatment is a cost-effective and environmentally friendly way to treat wastewater by reducing textile dye pollutants by dissolving dye molecules into less hazardous forms.	(10)
Microwave-assisted glycolysis of polyester	With the help of microwaves, ZnO-catalyzed fast polyester depolymerization speeds up the conversion of polyester into monomers. Reducing reaction times and encouraging material reuse in a sustainable circular economy are two ways that this effective procedure supports recycling.	(4)
Enzymatic hydrolysis of cotton-derived waste	By making materials more accessible, efficient pretreatment enhances enzymatic operations and makes it possible to produce high-value compounds from waste more sustainably and efficiently.	(2)
Fungal fermentation for cellulase production	Cotton and PET materials are used in fermentation operations to effectively manufacture cellulase, an enzyme used in industry that is created when microorganisms break down fibers.	(10)
Hydrothermal carbonization for biowaste	Sustainable waste management and resource use are supported by biowaste treatment, which makes it possible to remove toxins and recover valuable resources.	(1)
Thermochemical pyrolysis for textile waste	Using pyrolysis, textile waste may be recycled sustainably by producing energy and reusable materials. The necessity for specialized infrastructure, high costs, and scalability issues, however, make it economically unfeasible and necessitate improvements in cost-effectiveness and efficiency.	(9)

## 2 Chemical Techniques for Textile Waste Valorization

### 2.1 Hydrolysis

**Figure 1:**
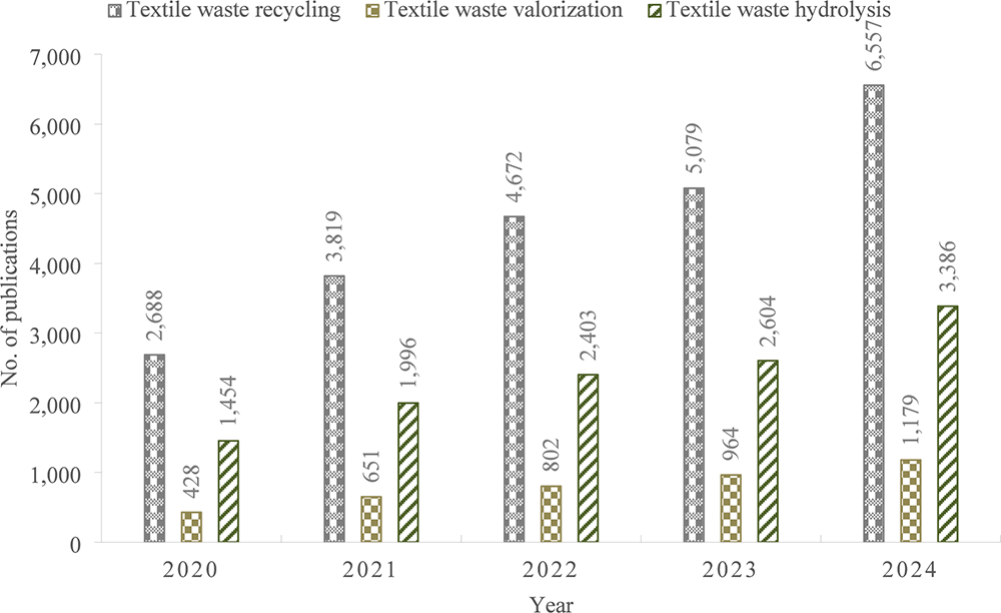
Publications trend of textile waste treatment (presented data based on the ScienceDirect database from 2020 to 2024).

Hydrolysis has emerged as a key technique in the valorization of textile waste due to its capacity to decompose complex textile polymers into simpler monomeric units.^30^ The growing interest in this field is evident from the increasing number of publications related to textile waste valorization and hydrolysis, which can be seen in Figure 1. This process is particularly essential for recycling blended fabrics, which often combine natural and synthetic fibers, complicating their separation and reuse.^31^ Hydrolysis, through acid, alkaline, or enzymatic approaches, offers efficient solutions for breaking down both cellulosic and synthetic materials. Each method operates under distinct chemical conditions, leading to varying degrees of efficiency depending on the textile type and its end-use.^32^ These processes play a critical role in overcoming challenges in recycling cotton, polyester, and blended fabrics by recovering resources such as glucose, terephthalic acid, and ethylene glycol.^30^

#### 2.1.1 Acid Hydrolysis

**Figure 2:**
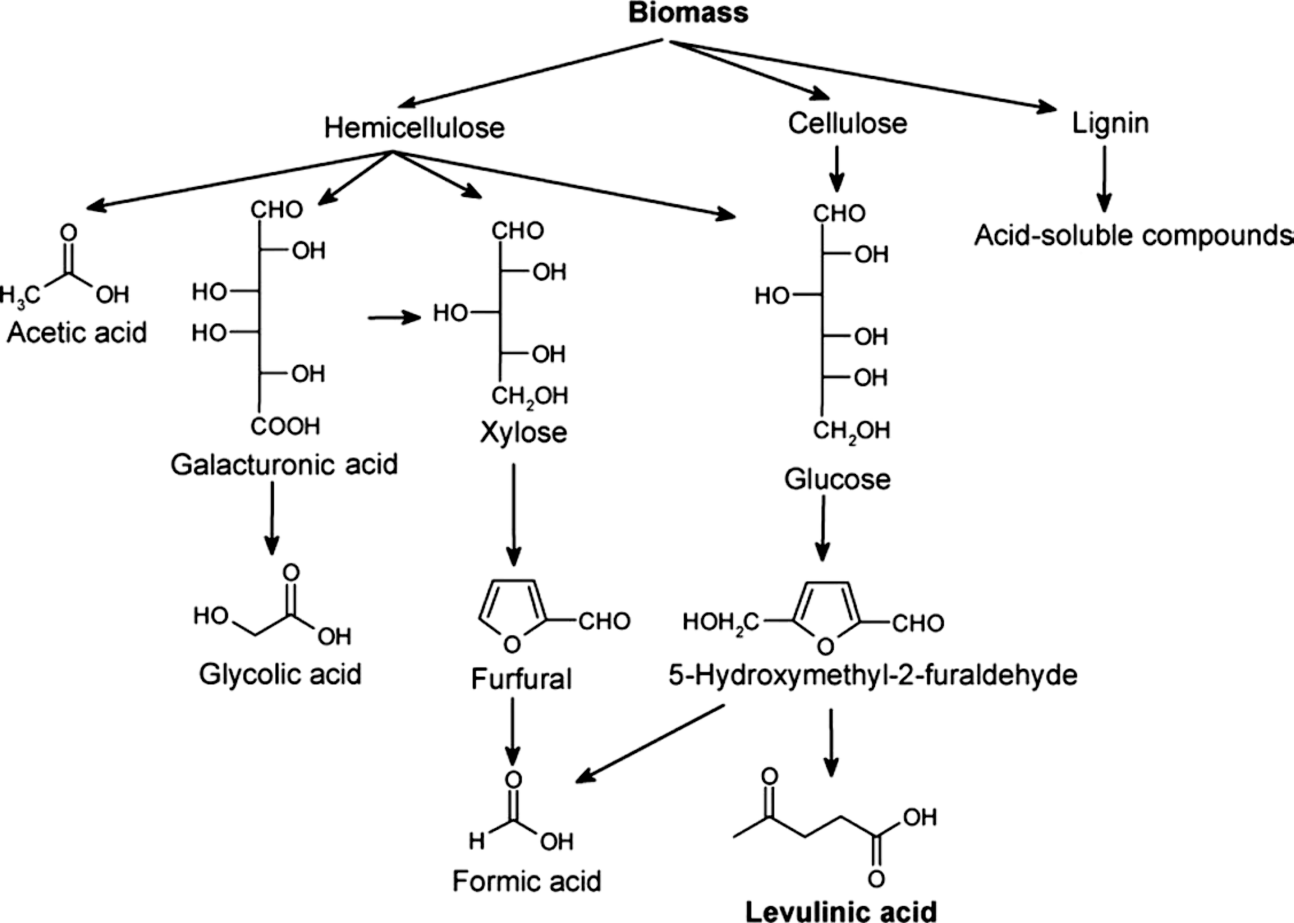
Possible reaction pathways for biomass acid hydrolysis. Reprinted with permission from ref (37). Copyright 2006 Elsevier Ltd.

Acid hydrolysis is a widely researched method used to break down cellulose into glucose, particularly effective for cotton-based textiles due to their high cellulose content. In this process, strong acids like sulfuric acid (H_2_SO_4_) are used to cleave the β-(1→4)-glycosidic bonds between glucose units, leading to the release of fermentable sugars such as glucose. Cotton, being composed of over 90% cellulose, is a prime candidate for this process.^33^ The hydrolysis occurs at high temperatures, typically between 120 and 160 °C, and with concentrated acid (up to 70%), conditions that are necessary to achieve the effective breakdown of the cellulose polymer into its glucose monomers.^34^ The reaction can be represented as follows:
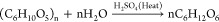
1Eq 1 describes how cellulose is hydrolyzed into glucose through the addition of water, facilitated by strong acid. After the hydrolysis, the acid must be neutralized, typically using bases like calcium hydroxide Ca(OH)_2_.^35^ The neutralization process is essential to control unwanted byproducts. Without neutralization, acidic conditions create harmful byproducts when glucose breaks down into furfural and hydroxymethylfurfural (HMF). These byproducts then further decompose to produce additional byproducts including formic acid, levulinic acid, and humins. The biomass breakdown process is shown in the Figure 2.^36^

An eco-friendly way to neutralize sulfuric acid in textile industry wastewater is by using carbon dioxide (CO_2_). When CO_2_ is added to the wastewater, it forms carbonic acid, which reacts with sulfuric acid and neutralizes it without adding harmful chemicals. This method also repurposes CO_2_, reducing its contribution to greenhouse gas emissions. Another approach uses bacteria to break down sulfuric acid, creating alkaline substances like ammonia that help balance the pH. Both methods are effective in minimizing environmental impact and promoting sustainable wastewater management.^38^ One of the key advantages of acid hydrolysis is its high efficiency, with glucose yields exceeding 70% under optimized conditions.^39^ This makes it a highly attractive option for converting textile waste into bioethanol, which can serve as a renewable energy source. However, challenges remain in managing the environmental impact of the acid waste, as large volumes of neutralized acid need to be handled and disposed of carefully.^40^ Additionally, the high crystallinity of cellulose in some textiles, particularly in polyester–cotton blends, presents a barrier to complete hydrolysis.^41^ This crystallinity makes the material more resistant to acid attack and thus limits the efficiency of the hydrolysis process. While acid hydrolysis is an effective method for breaking down cotton textiles into fermentable sugars, the process must be carefully managed to handle both the crystallinity of the material and the environmental impact of acid waste.^42^

#### 2.1.2 Base Hydrolysis

Base hydrolysis, also known as alkaline hydrolysis, is an effective process for breaking down synthetic fibers, particularly polyester (PET), which is widely found in textile waste. This process uses strong bases such as sodium hydroxide (NaOH) to cleave the ester bonds in PET, converting it into its constituent monomers, terephthalic acid (TPA) and ethylene glycol (EG).^35^ These monomers can be recovered and purified, making them suitable for synthesizing new PET in a closed-loop recycling system. The chemical reaction for the hydrolysis of PET is represented in eq 2.
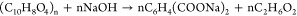
2This equation shows how PET, in the presence of NaOH, breaks down into disodium terephthalate and ethylene glycol.^43^ The disodium terephthalate can be further acidified to recover pure terephthalic acid (TPA).^34^ Base hydrolysis is often employed in the recycling of polycotton blends, where the polyester component is selectively depolymerized, leaving the cotton fibers intact.^35^ This selective breakdown allows for easier separation of the two materials, making it an efficient recycling method for blended textiles. A key advantage of base hydrolysis over acid hydrolysis is that it can be carried out under milder conditions, with lower temperatures and less hazardous byproducts.^42^ However, one of the challenges faced during the process is the interference from additives, dyes, and finishing chemicals present in the textile fibers. These substances can affect the efficiency of hydrolysis and the purity of the recovered monomers.^41^ In summary, base hydrolysis provides an effective and relatively environmentally friendly way to recycle polyester textiles, contributing to sustainable recycling practices by enabling the recovery of valuable monomers for the production of new PET.^42^

#### 2.1.3 Enzymatic Hydrolysis

**Figure 3:**
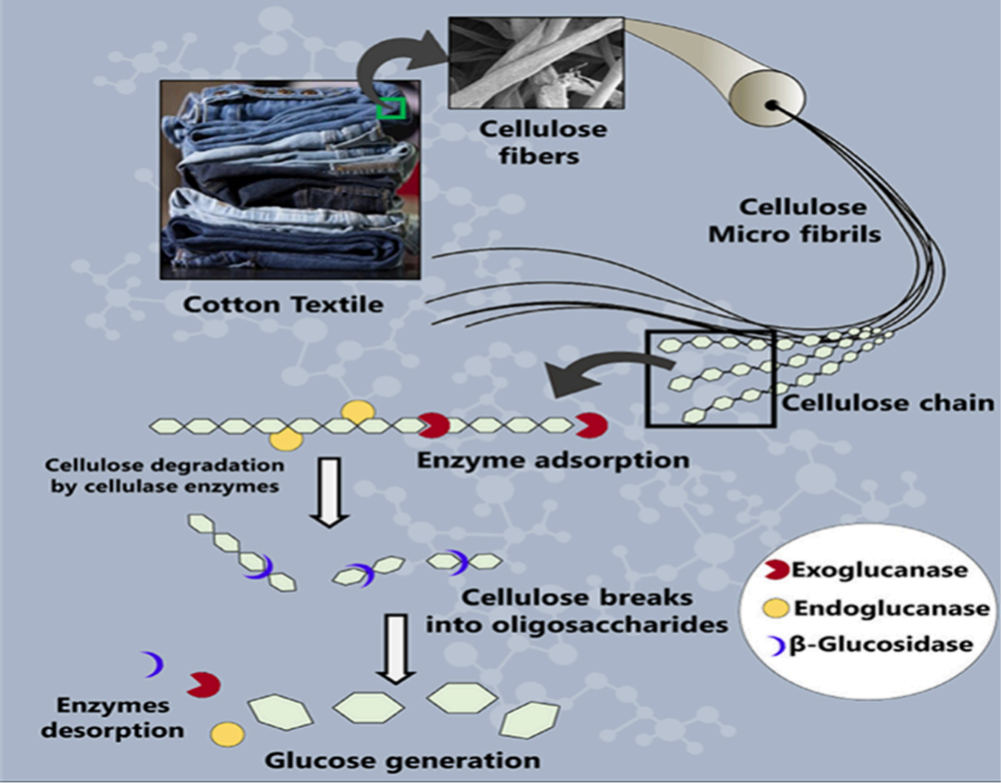
Schematic representation of the mechanism of cellulose enzymatic hydrolysis. Reprinted with permission from ref (23). Copyright 2022 Elsevier Ltd.

Enzymatic hydrolysis presents an environmentally friendly alternative to chemical methods, relying on enzymes such as cellulases, lipases, or cutinizes to degrade textile polymers under mild conditions.^43^ This method is particularly effective for cellulose-based textiles like cotton, where cellulases break down cellulose into glucose, which can then be fermented into biofuels or biochemicals as shown in Figure 3.^33^ The general enzymatic hydrolysis reaction of cellulose is represented in eq 3.
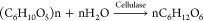
3This reaction demonstrates the conversion of cellulose into glucose through the action of cellulase.^44^ Enzymatic hydrolysis also plays a role in degrading synthetic polymers like polyethylene terephthalate (PET), though this requires specific enzymes such as cutinizes. These enzymes catalyze the breakdown of the ester bonds in PET, yielding terephthalic acid (TPA) and ethylene glycol (EG) as the end products. The use of cutinizes for PET hydrolysis offers an eco-friendly recycling approach, particularly for textiles containing PET blends.^42^ One of the most significant advantages of enzymatic hydrolysis is its operation under mild conditions, typically at neutral pH and moderate temperatures, which not only reduces energy consumption but also minimizes environmental impact.^34^ This is in stark contrast to the harsher conditions required in acid or base hydrolysis. Furthermore, enzymatic processes are highly specific, allowing for the selective breakdown of one component in blended fabrics without affecting others. For instance, enzymes can selectively hydrolyze the cellulose in a cotton–polyester blend, leaving the polyester intact, and thereby simplifying the recovery of both materials.^42^

Enzymatic and chemical hydrolysis differ significantly in energy efficiency, environmental impact, and scalability. Enzymatic hydrolysis operates under mild conditions, such as moderate temperatures and neutral pH, consuming less energy and producing minimal toxic byproducts, making it environmentally friendly. Despite these advantages, its scalability is limited by the high cost of enzymes, though advancements in enzyme recovery are improving feasibility.^34^ In contrast, chemical hydrolysis requires harsh conditions, such as high temperatures and strong acids or bases, leading to higher energy demands and hazardous byproducts. While chemical methods are well-established for large-scale operations, their environmental costs and infrastructure requirements pose significant challenges to sustainability.^45^ Nevertheless, ongoing research into enzyme optimization and industrial scalability holds promise for the broader adoption of this method. Enzymatic hydrolysis offers a sustainable and selective method for textile recycling, with applications for both natural and synthetic fibers, promoting circular economy principles by converting waste into valuable products.^35^

Enzyme recovery is crucial for reducing the environmental impact of manufacturing by minimizing energy and chemical use while decreasing waste. A widely used method is enzyme immobilization, which involves attaching enzymes to solid supports, creating a heterogeneous immobilized enzyme system.^46^ This system mimics the natural state of enzymes in living cells, where they attach to structures like the cytoskeleton, membranes, and organelles. Immobilization stabilizes enzyme structure, enhancing its activity, robustness, and resistance to environmental changes compared to free enzymes. Additionally, immobilized enzymes enable easier recovery, multiple reuse, continuous processes, rapid reaction termination, and greater flexibility in bioreactor designs.^47^ Similarly, membrane filtration methods, such as ultrafiltration, are effective for separating and concentrating enzymes for reuse, significantly lowering costs in industrial applications. Ultrafiltration membrane technology concentrates proteins and recovers enzymes, offering a cost-effective alternative to vacuum evaporation, especially for heat-sensitive enzymes. This approach ensures simultaneous concentration and purification with minimal activity loss, with dry methods available for later stages as needed. In Figure 3, another technique, centrifugal precipitation chromatography, uses agents like ammonium sulfate to recover enzymes cost-effectively at an industrial scale. Combining counter-current chromatography with protein precipitation leverages the differing solubility of proteins in ammonium sulfate.^48^ These advancements enhance the feasibility and affordability of enzymatic hydrolysis for large-scale applications, including textile waste valorization.

### 2.2 Pyrolysis

Pyrolysis is a thermal decomposition process in which organic materials are subjected to heat in the absence of oxygen, leading to the breakdown of their chemical bonds. The process occurs in multiple stages, starting with the removal of moisture and followed by the degradation of polymers such as cellulose and lignin. As the temperature increases, the material is converted into three primary products: gases, liquid bio-oils, and solid char. At lower temperatures, pyrolysis tends to yield more bio-oils, while higher temperatures favor the formation of gases and char.^49^ Unlike combustion and gasification, pyrolysis takes place in the absence of oxygen, allowing for controlled thermal decomposition, making it a versatile technique for processing organic waste. The products generated-gases, oils, and char have a range of applications, including energy production, chemical feedstock, and soil enhancement. The efficiency of the process and the distribution of products depend on factors such as the feedstock type and reactor conditions like temperature and heating rate.^50^

**Figure 4:**
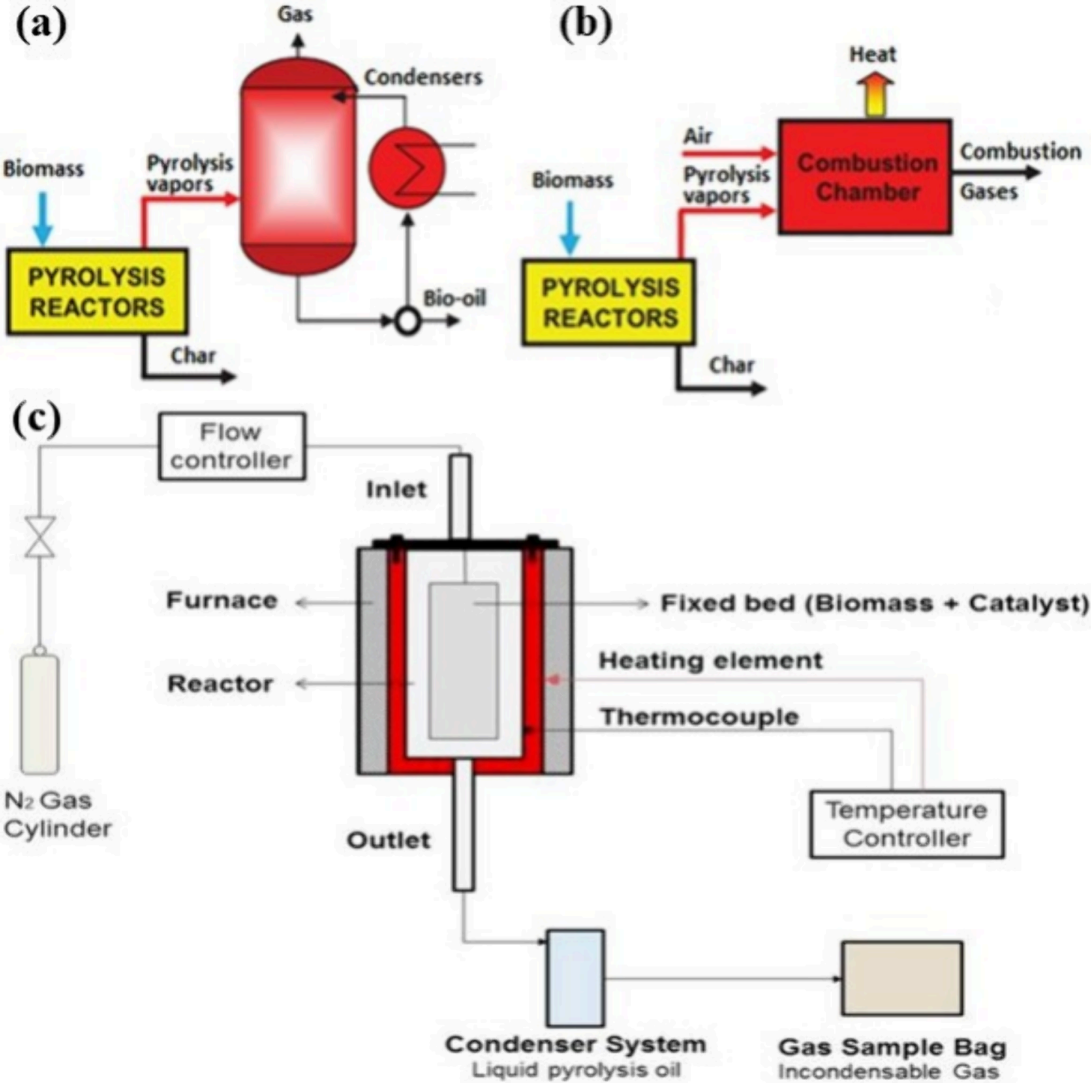
Flow diagrams for simplified pyrolysis of biochar and bio-oil (a) and biochar and gas (b). Reprinted with permission from ref (52). Copyright 2017 Intech. Schematic diagram of the in situ fixed bed pyrolysis setup (c). Reprinted with permission from ref (53). Copyright 2017 Elsevier Ltd.

Pyrolysis systems are generally designed in two configurations. The first configuration consists of a reactor, a distillation column, a condenser, and a reboiler, as depicted in Figure 4a. This setup produces a combination of liquid products, solid char, and gases. The second configuration, shown in Figure 4b, includes a reactor and a combustion chamber. Unlike the first, this arrangement does not yield liquid bio-oil due to the absence of a condenser. Instead, the feed is entirely thermally decomposed into gases, which are then used in the combustion chamber as a heat source.^51^

Pyrolysis processes are divided into three main categories, as shown in Table 2, with variations in reaction temperature, heating rate, feedstock size, and residence time. These operating conditions greatly affect the types of products generated. For example, fast pyrolysis, which utilizes finely ground feedstock, produces a high yield of bio-oil, whereas slow pyrolysis, using whole feedstock, results in a lower bio-oil output. Adjusting these parameters allows for tailoring the product composition based on the feedstock used.^54^

**Table 2:** A Summary of the Different Types of Pyrolysis Processes and Their Operating Parameters^a^

Process	Reaction temp (°C)	Heating rate (°C/min)	Residence time	Feedstock size	Bio-oil yield (%)	Biochar yield (%)	Gas yield (%)
Slow pyrolysis	300–550	50	5–30 min: 25–35 h	Whole	20–50	20–35	20–50
Intermediate pyrolysis	300–450	200	10 min	Coarse/finely ground	35–50	25–40	20–30
Fast/flash	300–1000	10–1000	2 s	Finely ground	60–75	10–25	10–30

a Reprinted with permission ref (54). Copyright 2018 Elsevier Ltd.

The progression of the pyrolysis process is influenced by specific operating conditions. Initially, the removal of moisture is essential before chemical breakdown can occur. Once dehydration is complete, polymers like cellulose and lignin degrade into simpler compounds, which eventually evolve into gases, bio-oils, or char depending on the conditions.^49^ The feedstock type, reactor temperature, and heating rate are key factors that influence how these reactions progress.^55^ The rate of pyrolysis, as well as the yield of gases, oils, and char, is closely linked to the temperature and the speed at which heat is applied. At lower temperatures, typically between 300 and 500 °C, bio-oils are the dominant product. These oils can be processed further into fuels or chemicals. However, at these moderate temperatures, the material does not fully decompose, resulting in lower gas and char yields.^56^ As temperatures exceed 700 °C, gas production increases, and materials break down into lighter gases such as carbon monoxide, methane, and carbon dioxide.^57^ A larger portion of the material also converts into solid char, with its properties such as porosity and surface area depending on the feedstock and temperature, making it valuable for energy production and environmental remediation.^56^

Reactor design plays a critical role in pyrolysis efficiency and product distribution. Two common reactor types are fixed-bed and fluidized-bed systems. In fixed-bed reactors (Figure 4c), materials are heated at a controlled rate, resulting in the efficient separation of gases, oils, and char.^53^ This reactor is simple in design and cost-effective, producing lower bio-oil yields of approximately 43% and higher char yields of up to 33%, reflecting their limited efficiency.^58^

**Figure 5:**
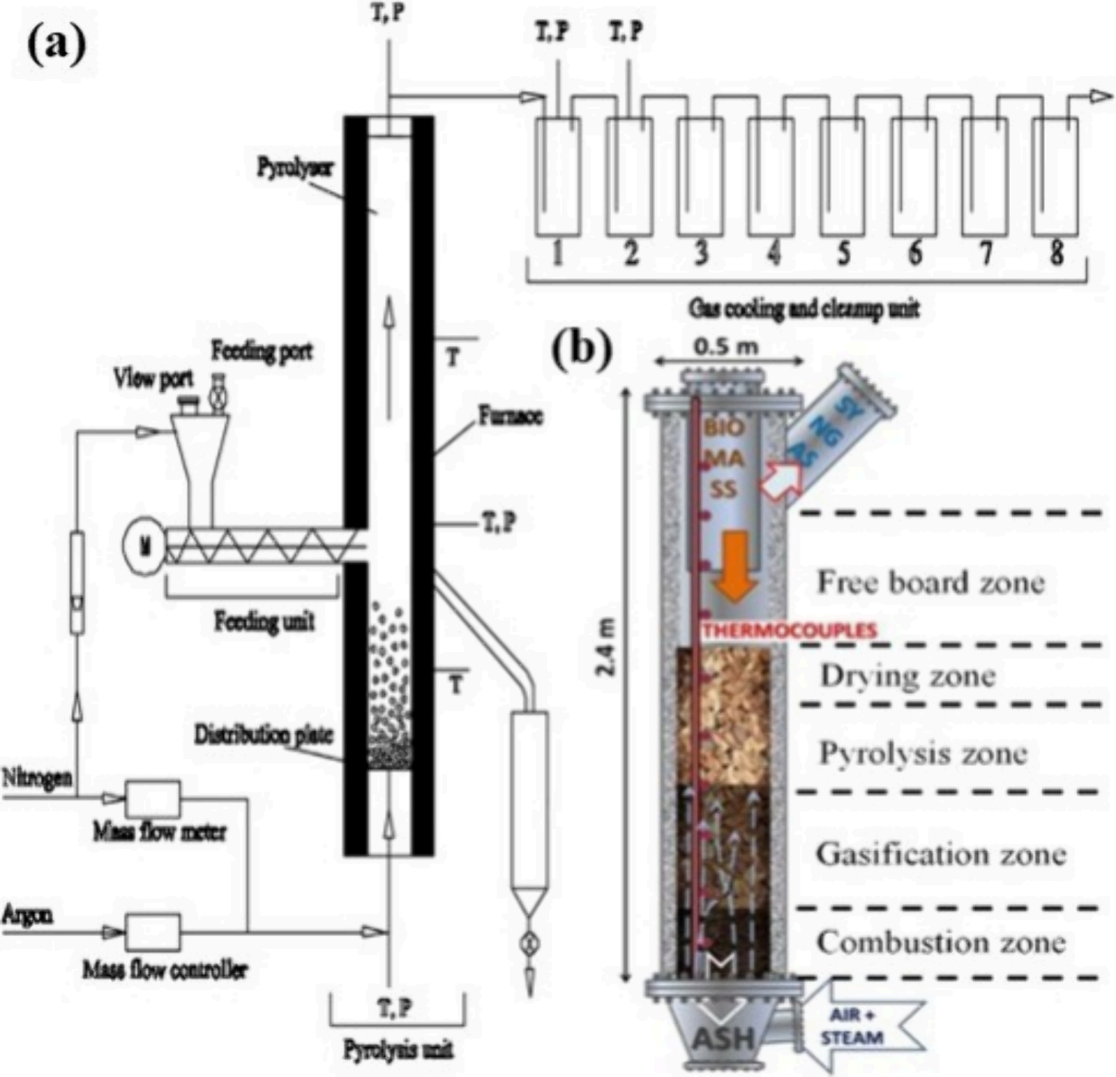
Experimental setup of the fluidized bed pyrolyzer (a). Reprinted with permission from ref (59). Copyright 2019 Elsevier Ltd. Experimental setup of the fluidized-bed gasifier systems (b). Reprinted with permission from ref (62). Copyright 2023 Elsevier Ltd.

The reactor design is often used for smaller-scale operations and tends to produce higher yields of char due to its steady heating conditions.^50^ In contrast, fluidized-bed reactors provide uniform heating, allowing for higher reaction rates and optimized production of bio-oils and gases, making them more suitable for large-scale, continuous processes (Figure 5a).^59^ This reactor can achieve enhanced heat transfer rates due to solid–solid interactions, allowing for more uniform temperature distribution. Operating at temperatures ranging from 450 to 520 °C, it exhibits continuous operation and bio-oil yields of up to 60%, making it suitable for large-scale applications.^57^ Additionally, fluidized-bed systems benefit from reduced operational issues, such as coke buildup, due to better mixing of feedstock.^60^ Contrarily, fixed-bed reactors face challenges in scalability and require longer residence times for feedstock conversion, limiting their industrial application.^61^

**Figure 6:**
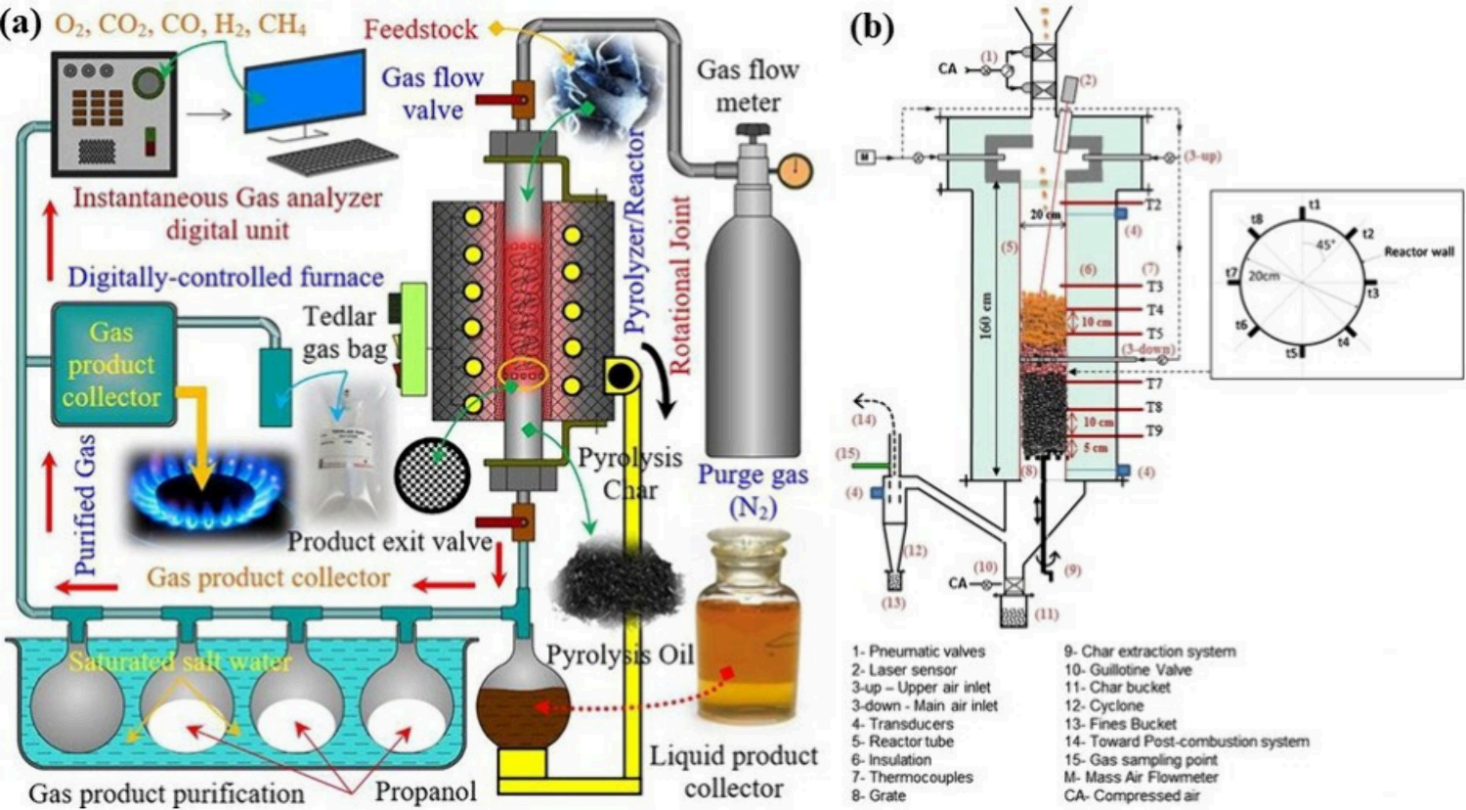
Pyrolysis process for textile waste valorization into bio-oil and gas products (a). Reprinted with permission from ref (64). Copyright 2019 Elsevier Ltd. Schematic representation of the downdraft reactor (b). Reprinted with permission from ref (65). Copyright 2019 Elsevier Ltd.

An advanced method called steam pyrolysis incorporates steam into the process to enhance material breakdown. Steam acts as a mild oxidizing agent, promoting the cracking of heavier hydrocarbons and increasing gas yields, particularly hydrogen. This method is beneficial when the goal is to maximize gas production while minimizing tar formation and improving char quality.^63^ Steam pyrolysis can be used in both fixed-bed and fluidized-bed reactors to enhance gas production, especially for lighter gases like hydrogen, and reduce tar production, making the process more flexible for various applications.^50^ Pyrolysis has proven to be an effective solution for managing textile waste by converting discarded fibers into valuable products like biochar, bio-oils, and gases. Conventional disposal methods, such as incineration or landfilling, contribute to environmental harm, but pyrolysis offers a cleaner alternative. Natural fibers like cotton decompose into biochar, which can be used for soil improvement and environmental remediation, while synthetic fibers such as polyester break down into fuel-grade oils and gases.^56^Figure 6a depicts the pyrolysis process for transforming textile waste, such as old jeans, into valuable energy carriers like bio-oil and gases like methane and hydrogen. The setup consists of a pyrolysis reactor, a gas purification unit, and an instantaneous gas analysis system, which demonstrate the effective conversion of over 82% of the feedstock into liquid and gas products. This process demonstrates pyrolysis’s promise as a sustainable means of valorizing textile waste by optimizing energy recovery while minimizing reaction time.^64^

### 2.3 Gasification

Gasification is a thermochemical process that converts carbon-based materials, such as textile waste, into syngas by exposing them to high temperatures in an oxygen-limited environment.^66^ Unlike combustion, gasification occurs with a controlled oxygen supply, typically less than required for complete combustion.^67^ This results in the production of syngas, a mixture primarily composed of hydrogen (H_2_), carbon monoxide (CO), carbon dioxide (CO_2_), and methane (CH_4_).^65^ Gasification technology has gained significant attention as a method for converting waste materials, including textiles, into usable energy, while reducing landfill waste and environmental pollution.^68^ Various types of reactors are used for gasification, with fluidized bed reactors being among the most efficient for waste-to-energy applications (Figure 5b). These reactors are known for their ability to handle large volumes of waste and ensure thorough mixing of the feedstock with the gasifying agents, improving the overall conversion process.^69^ The syngas produced through gasification can be used in various applications, such as electricity generation, heating, or as a chemical feedstock.^69^ Another commonly used reactor is the downdraft gasifier, which is favored for its ability to produce cleaner syngas by reducing tar formation as the gas moves through high-temperature zones.^65^

Textile waste, including natural fibers like cotton and synthetic fibers like polyester, presents an opportunity for gasification.^70^ Synthetic fibers, such as polyester, produce hydrogen-rich syngas, increasing the energy content of the fuel.^71^ Natural fibers, like cotton, produce carbon-based gases, adding diversity to the syngas composition.^69^ As the global accumulation of textile waste increases, particularly from fast fashion, gasification offers a sustainable solution to reduce waste and recover energy.^72^ Additionally, updraft gasifiers are also used in some textile waste gasification applications, particularly when operating with mixed feedstocks, as they provide an efficient way to gasify various materials in a vertical flow design (Figure 6b).^73^

Exhibiting noteworthy projects like Air Products and Chemical’s facility in Port Arthur, Texas, which uses petcoke and heavy crude oil residues to produce hydrogen and carbon monoxide while integrating carbon capture technology for low emissions, industrial-scale gasification implementations have gained traction across various sectors for converting diverse feedstocks into syngas.^74^ Shell’s Pearl GTL plant in Qatar uses Fischer–Tropsch synthesis to turn natural gas into premium liquid hydrocarbons, such as diesel. After overcoming early operating difficulties, Duke Electric’s IGCC power plant in Indiana uses coal to produce cleaner electricity and gradually increases efficiency.^75^ In order to reduce greenhouse gas emissions, World Energy’s biorefinery in California uses the All-Cellulose Technology platform to transform agricultural leftovers into renewable biofuels.^76^ In order to promote cleaner vehicle fuel projects, Nexterra Systems Corp. in Canada concentrates on biomass power generation from wood waste, while Gasrec in the UK employs anaerobic digestion to produce biomethane. General observations emphasize the necessity of quality control for optimal output quality, the flexibility in adapting feedstocks to market situations, and the growing integration of carbon capture technology to reduce net emissions throughout various projects.^77,78^

A list of reactions or the gasification process is given in Table 3, where syngas is produced through chemical reactions including partial oxidation, steam reforming, and water–gas shift reactions. A key reaction is the water–gas shift reaction: C + H_2_O → CO + H_2_.^79^ The syngas is rich in hydrogen and carbon monoxide, both of which are combustible gases with high energy potential. In terms of syngas composition, the amounts of CO and H_2_ in syngas are important for downstream uses such as Fischer–Tropsch processes, methanol synthesis, and synthesis gas production. The ratios of these gases produced by various feedstocks vary. Changes in the feedstock may cause the syngas to contain contaminants such as sulfur compounds, nitrogen species, or particles. These contaminants necessitate further purifying procedures and may impact catalyst performance.^80,81^ The chemical makeup of biomass, coal, and organic waste varies, which affects how much volatiles are generated during gasification or pyrolysis. For example, biomass is more suited for operations that require hydrogen since it usually produces a larger percentage of H_2_ than coal.^82^ For the production of clean fuels using Fischer–Tropsch synthesis or methanation, a higher H_2_ content is preferred. For some chemical syntheses, specific ratios (such as a 2:1 ratio of H_2_ to CO) are ideal; thus, the feedstock selection must be in line with process specifications. Higher CO content syngas might be more suitable for internal combustion engine or gas turbine combustion. Variability can result in uneven syngas quality, which makes process optimization and control more difficult. Variations in feedstock quality call for flexible processing technologies that can adjust to variations without suffering appreciable efficiency losses. Decisions about which materials are used for the manufacture of syngas are influenced by the economic viability of specific feedstocks, which is directly related to their market price and geographic availability.^80^ So, the composition of syngas can vary depending on the type of feedstock, operating conditions, and gasifying agents used.^71^

**Table 3:** Reactions during Gasification^a^

Sl. no.	Reaction name	Reactions	Δ*H* (MJ/kmol)
1	Combustion	C + O_2_ → CO_2_	–394.3
2	Partial combustion	C + 0.5O → CO	–110.5
3	Boudouard	C + CO_2_ → 2CO	172.5
4	C_n_H_m_ Partial combustion	C_n_H_m_ + 0.5O_2_ → nCO + 0.5_m_H_2_	Exothermic
5	Water–gas primary	C + H_2_O → CO + H_2_	131.3
6	Water–gas secondary	C + 2H_2_O → CO_2_ + 2H_2_	90.2
7	Water–gas shift	CO + H_2_O → CO_2_ + H_2_	–41.2
8	Reverse water–gas shift reactions	CO_2_ + H_2_ → CO + H_2_O	41.2
9	Steam reforming	C_n_H_m_ + nH_2_O → nCO + (n + 0.5m)H_2_	Exothermic
10	Dry reforming	CH_4_ + CO_2_ → 2CO + 2H_2_	Exothermic
11	Methane reforming	CH_4_ + H_2_O → CO + 3H_2_	206.2
12	Methanation	CO + 3H_2_ → CH_4_ + H_2_O	–206.2
13	Carbon monoxide combustion	CO + 0.5O_2_ → CO_2_	–283.4

a Reprinted with permission from ref (69). Copyright 2023 Elsevier Ltd.

Gasification of textile waste not only helps to reduce landfill burden but also mitigates environmental impacts like methane emissions and leachate formation. Synthetic polymers such as polyester, which have high calorific values, are especially well-suited for gasification as they produce energy-dense gases such as hydrogen and carbon monoxide.^83^ These gases can be cleaned of impurities and used for power generation or as feedstocks for producing synthetic fuels.^84^ Compared to incineration, gasification operates in a controlled environment that reduces the release of harmful emissions like dioxins, furans, and nitrogen oxides (NOx).^69^ Additionally, syngas produced through gasification can be purified of contaminants like sulfur and tar, making it suitable for a range of industrial applications including electricity generation and chemical processes.^65^ Economically, gasification enables energy recovery from textile waste that would otherwise be sent to landfills, reducing reliance on fossil fuels.^70^ Integrating gasification into waste management systems supports a circular economy, in which waste is continuously recycled and energy is recovered sustainably.^85^

### 2.4 Aminolysis and Glycolysis

Through the breakdown of synthetic fibers like polyester into high-purity monomers that may be recycled to make new materials, textile waste valorization via aminolysis presents a viable alternative to lessen reliance on landfills. Using life cycle assessment guarantees that aminolysis technologies are evaluated methodically and in accordance with the economic, social, and environmental requirements for incorporation into the regional circular economy.^86^

For textile waste valorization, glycolysis is a potential chemical recycling technique. It breaks down polyethylene terephthalate (PET) fibers into monomers like bis(2-hydroxyethyl) terephthalate (BHET), which can then be used to create new materials. With this method, PET fabrics’ structural variations and additives are addressed, providing a more sophisticated option than mechanical recycling. The textile sector can support environmentally friendly PET recycling by optimizing glycolysis to meet sustainable development goals by assessing green metrics like energy-saving coefficients and sustainable catalysts.^87^

Polyester (PES) textile fibers can be chemically recycled into high-purity monomers like bis(2-hydroxyethyl) terephthalate (BHET) and bis(2-hydroxyethyl) terephthalamide (BHETA) by using aminolysis and glycolysis, which are catalyzed by In-ZnO/g-C_3_N_4_ nanocomposites. The wet-impregnation approach produced nanocomposites that exhibit improved catalytic performance because of their altered optical characteristics. In addition to enabling the sustainable recycling of PES textiles into reusable monomers, this method efficiently eliminates dyes and contaminants, improving the management of textile waste. The effectiveness and purity of the final monomers are verified by a number of characterization methods.^88^

### 2.5 Hydrothermal Processing

**Figure 7:**
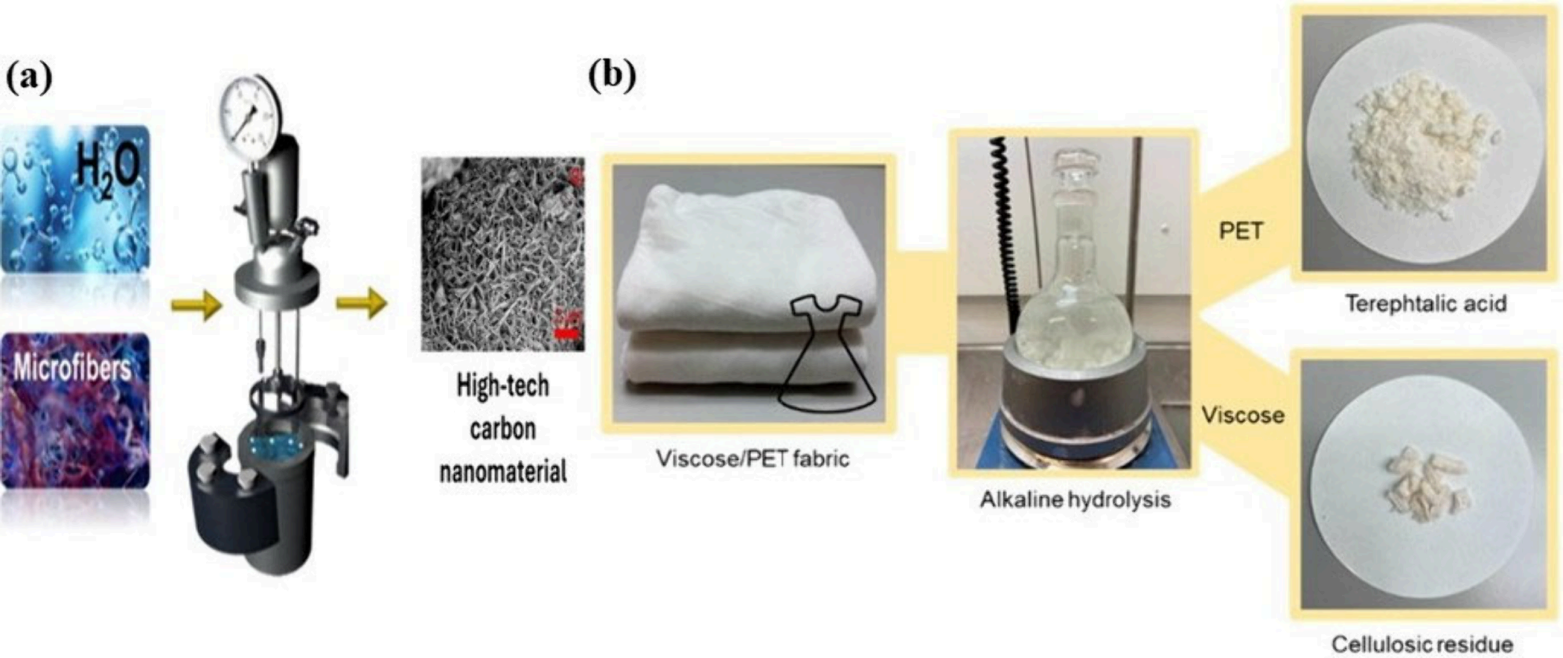
Hydrothermal carbonization process for converting microfiber waste into carbon nanomaterials (a). Reprinted with permission from ref (90). Copyright 2024 iScience. Schematic of the hydrolysis reaction (b). Reprinted with permission from ref (91). Copyright 2022 MDPI.

Hydrothermal waste valorization efficiently turns cotton waste, like towels, into glucose and useful compounds by using direct hydrolysis and microwave-assisted treatment. Enhanced by sulfuric acid as a catalyst, the method gives high glucose yields and valuable byproducts such as levulinic acid and 5-hydroxymethylfurfural. Furthermore, ethanol synthesis is supported by the resulting glucose-rich supernatant, demonstrating a sustainable method for converting textile waste into biobased chemicals and fuels.^89^ The process of hydrothermal carbonization (HTC), which turns waste synthetic microfibre into high-value carbon nanoparticles, is depicted in Figure 7a. Under regulated circumstances, cotton and polyester microfibers are transformed into amorphous and graphitic carbon structures, such as carbon nanotubes, using a Fe–Ni catalyst. Through the production of sophisticated carbon compounds with potential technological applications, this novel textile waste valorization technology integrates waste into the circular economy while addressing microfibre pollution in aquatic habitats.^90^

## 3 Affecting Factors in Chemical Valorization

### 3.1 Textile Composition

The unique properties and difficulties presented by natural, synthetic, and blended fibers make the composition of textile materials an important consideration in the chemical valorization process. Since cellulose makes up the majority of natural fibers like cotton, they are ideal for enzymatic hydrolysis and biorefining to produce fermentable sugars that can be used to produce biofuel. Their high cellulose content makes it simpler to convert them into goods with additional value, such biofuels and biochemicals. On the other hand, synthetic fibers like as polyester, although robust, cannot break down naturally and must be broken down into recyclable monomers using chemical and enzymatic techniques. Blended fibers pose unique challenges since they contain both synthetic and natural elements. To efficiently recover cellulose and polyester, separation procedures such as enzymatic hydrolysis and ionic solutions are required. All things considered, the particular makeup of textiles has a major impact on the tactics chosen for valorization, which are meant to improve sustainability by optimizing recovery and reducing environmental impact.^92^ Since viscose and polyester fibers have special qualities, different procedures are involved in the chemical valorization of textile materials. Hydrolysis is a particularly good method for cellulose, which includes viscose, to help turn it into useful goods such specialty chemicals and nanocrystalline cellulose. Terephthalic acid and ethylene glycol are produced during the alkaline hydrolysis of polyester (PET), which makes it recyclable. In blended fabrics in particular, the difficulty is in efficiently sorting and processing these fibers to guarantee full valorization (Figure 7b). This calls for ideal circumstances, including certain chemical concentrations and temperatures, to improve recycling processes’ sustainability and effectiveness.^93^

PET is broken down into monomers via the alkaline hydrolysis process, which reacts with sodium hydroxide to produce terephthalic acid in its sodium salt form and ethylene glycol and possible chemical reaction is presented in eq 4.
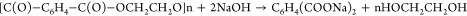
4This method effectively separates synthetic polyester from mixed materials. Meanwhile, the cellulose component of viscose remains intact as a solid cellulosic residue since it is neither chemically dissolved or hydrolyzed under these alkaline conditions. This approach is essential for isolating and valorizing PET and viscose components.

### 3.2 Additives and Contaminants

Textile processing involves the use of about 8000 chemicals, including coatings, finishes, softness, dyes, and mordants. The ability of recycling fabrics is significantly limited by the presence of such substances.^94^ Technology makes it possible to recycle PET bottles, but because polyester garments include colors, the same process cannot be used to recycle polyester fabrics. For example, the degree of polymerization during the melting process may be lowered if dispersion dyes are not removed from the polyester fabric before recycling.^95^ The deterioration process becomes more difficult due to the different kinds of chemicals used in the dyeing and finishing stages. Furthermore, there’s a developing market for functionalized and high-performance fabrics. Applying various chemicals as coatings is necessary to provide textiles unique properties like hydrophobicity and water resistance. The recycling process is made much more challenging by these unique finishes and coatings.^96^ Textile pollutants, such as dyes and finishes, can have a substantial effect on chemical valorization methods by impeding effective material recovery. Due to their resistance to biodegradation, many colors, such azo or reactive dyes, clog waste streams and make recycling procedures more difficult. Completing agents, such as water-repellents or antibacterial treatments, also add compounds that obstruct fiber breakdown and reprocessing. The efficiency of valorization techniques like hydrolysis and pyrolysis is decreased by this resistance to degradation, requiring extra preprocessing procedures to eliminate or neutralize these pollutants.^97^

### 3.3 Temperature

Variations in temperature have a major impact on the gasification of waste textiles, affecting the composition of the products, conversion efficiency, and gas output. larger temperatures (such as 850 °C) result in larger gas yields because the waste material is more effectively converted, generating more carbon dioxide, hydrogen, and carbon monoxide. For example, as temperature rises, polyester produces more hydrogen, and polymer breakdown also leads to the growth of aromatic chemicals like benzene. Natural fibers like cotton are perfect for generating syngas because they yield more syngas at higher temperatures and with less tar formation. Blends of cotton and polyester work well together (Figure 7a), and 750 °C is the ideal temperature to produce syngas and aromatic compounds such as BTXS (benzene, toluene, xylene, and styrene).^98^ Temperature has a big impact on how well chemical valorization procedures like pyrolysis work. Larger temperatures improve the quality of gases and oils by creating molecules with a larger energy content, whereas lower temperatures typically provide more value products like bio-oils. Temperature control is essential for maximizing the value of textile waste since rising temperatures alter the composition of the goods, forming more complex and energy-rich compounds.^99^

### 3.4 Pressure

Pressure plays an important function in chemical valorization, especially in procedures like hydrolysis. High pressure can improve the effectiveness of hydrolyzing polyester (PET) fibers into primary materials, like monomers and intermediates, in textile waste valorization without the need of dangerous chemicals. One efficient technique for separating and recovering components in multicomponent textile blends, such as cotton/polyester and wool/polyester, is high-pressure neutral hydrolysis.^100^ In addition to its role in hydrolysis, pressure is essential for improving gasification procedures for textile waste valorization. By applying increased pressure, gasification may convert textile waste more efficiently into syngas, a useful energy source. This is especially crucial in recent breakthroughs, where pressure-assisted gasification has resulted in increased clean energy outputs while lowering emissions of toxic byproducts. Furthermore, pressure plays an important role in processes such as solvolysis, where it improves solubility and reaction rates, enabling the valorization of mixed fiber textiles, such as cotton–polyester blends, more viable and sustainable. These developments highlight the role of pressure in boosting the economic and environmental feasibility of textile waste treatment methods.^100^

### 3.5 Catalyst Used

**Figure 8:**
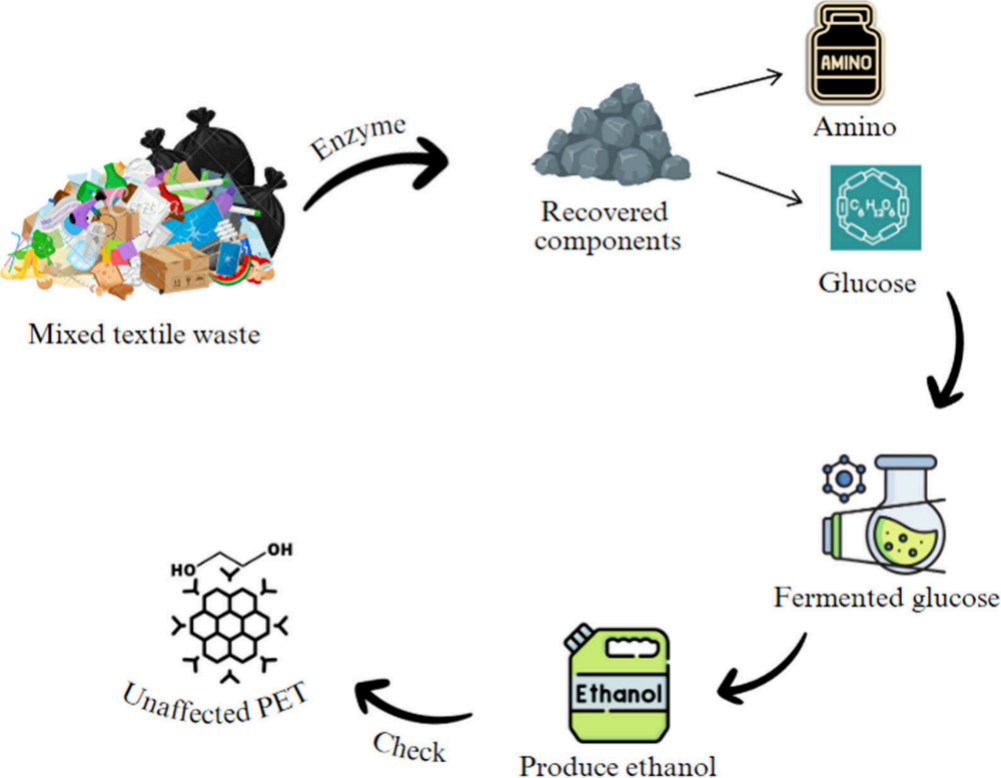
Enzyme-based catalysis for textile waste valorization.

Catalysts play an important role in increasing the efficiency and sustainability of textile waste valorization processes. The desire for more ecologically friendly solutions has resulted in a shift away from traditional chemical-based catalysts and toward greener, biobased alternatives. Enzyme-based catalysis, in particular, has received widespread interest for its ability to replace corrosive chemicals in textile manufacturing, providing both environmental and operational benefits.^101^ This enzyme-based method efficiently demonstrates the possibility of recovering valuable components from mixed textile waste, which contributes to circular economy concepts. By specifically targeting wool and cotton components with protease and cellulase enzymes, high-efficiency amino acid and glucose recovery was obtained, whereas PET fibers were unaffected (Figure 8). The recovered glucose was effectively fermented into ethanol, indicating that these recovered materials have the potential for additional industrial applications.^102^

The topic of textile waste valorization has seen a substantial transformation due to recent developments in metal and biobased catalysts, which provide sustainable methods for turning waste materials into useful goods. Because they are environmentally friendly and can function in mild environments, biobased catalysts—which are frequently made from natural sources like enzymes or plant extracts—have shown promise in the efficient breakdown of complex textile polymers without the use of harsh chemicals. This method improves product recovery rates while reducing the negative effects on the environment.^103,104^ However, metal catalysts, especially those that use transition metals like copper or palladium, are being developed more and more for their efficiency and selectivity in chemical reactions that turn textile waste into valuable chemicals or fuels. Through sophisticated synthesis methods, recent developments aim to decrease the toxicity of these metal catalysts while increasing their stability and recyclability. In order to maximize reaction pathways and attain greater yields in the valorization process, both types of catalysts are increasingly being integrated into hybrid systems. This promotes a circular economy in the textile sector.^105,106^ Furthermore, research into catalyst regeneration has extended the life of metal catalysts by tackling challenges such as coke deposition and metal sintering, allowing for more sustainable, recurrent use. By extending the life of current catalysts, catalyst regeneration lessens the requirement for new raw materials in an environmentally friendly manner. This promotes a more sustainable production process by reducing the amount of resources extracted and the waste produced during the disposal of spent catalysts.^107^ Furthermore, recycling catalysts reduces the energy needed for their manufacture and development; revitalizing rather than replacing existing materials uses less energy. Reusing catalysts can save manufacturers a significant amount of money. Regeneration techniques that enable continued use without the need for frequent replacements might gradually balance the initial expenditure on high-quality catalysts.^108^ Overall, these innovations are pushing the frontiers of textile waste use by reducing energy consumption and enhancing the overall economic viability of metal-catalyzed processes.^102^

### 3.6 Solvent

In the solvolysis techniques used to extract fibers from blended textiles, especially when recovering polyester (PET) and elastane from cotton-based textiles, the choice of solvent is crucial. For example, methods like dissolution or hydrolysis can be used to separate polyester from cotton; the effectiveness of fiber breakdown depends on the chemical characteristics of the solvent. Selective recycling of individual fibers from complicated blends is made possible by the ability of effective solvent systems, such as those employed in enzymatic hydrolysis or acid-catalyzed hydrothermal treatments, to target certain polymers. The success of the recycling process is also influenced by the solvent’s compatibility with different additives, such as dyes and finishes. Selecting the appropriate solvent is crucial for improving the sustainability of the recycling process as well as the purity of recovered fibers.^109^ The choice of solvent has a direct effect on the effectiveness and purity of fiber recovery in procedures like the chemical recycling of blended textiles. To prevent harming cotton or other natural fibers in the blend, for example, the solvents used to depolymerise polyester must be carefully chosen. To reduce contamination and improve the quality of the recovered fibers, solvent compatibility with additives like dyes and finishes is also essential. In textile waste valorization, solvent recovery and reuse are also crucial factors to take into account in order to lessen the impact on the environment and operating expenses. Therefore, choosing a solvent in a balanced way not only enhances recycling results but also supports waste management sustainability objectives.^109^

### 3.7 Pretreatment

**Figure 9:**
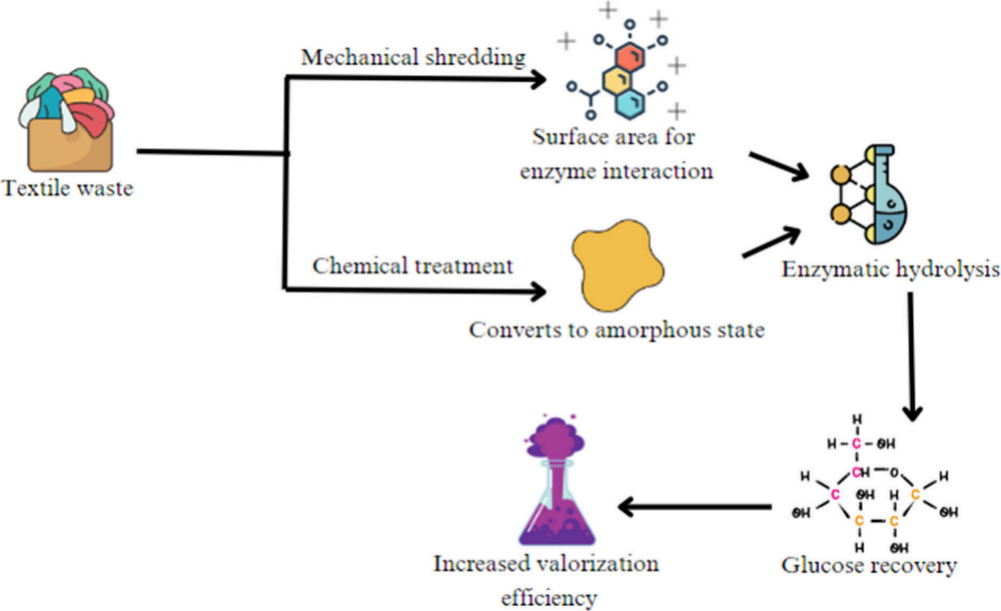
Mechanical and chemical pretreatments of textile waste valorization.

Pretreatment is an important stage in textile waste valorization since it prepares the material for later recycling operations. Pretreatment of textile waste frequently involves mechanical, chemical, or thermal processes to remove contaminants such as dyes and finishes, as well as break down fiber structure. Proper pretreatment improves the effectiveness of enzymatic hydrolysis by making fiber components more accessible to the enzymes or chemical agents utilized. This phase is critical for effectively recovering precious fibers and producing viable value-added products from textile waste.^92^ To maximize the efficiency of enzymatic hydrolysis in textile waste valorization, mechanical and chemical pretreatments must be combined. Mechanical shredding shrinks fibers to a smaller size, increasing surface area for improved enzyme interaction and chemical treatments, such as sodium hydroxide, are also employed to diminish cellulose crystallinity and convert it to an amorphous state, which improves enzyme accessibility (Figure 9). The combination of mechanical and chemical pretreatments considerably improves glucose recovery, with studies claiming yields of up to 90%. Proper pretreatment of textile waste not only speeds up fiber breakdown but also increases total valorization efficiency.^110^ Pretreatment also aids in the removal of dyes, finishes, and other impurities that may interfere with future recycling procedures. The kind and nature of textile waste determine the pretreatment process used, both natural and synthetic fibers require various treatments. Efficient pretreatment increases the recovery rate of valuable components while reducing the energy and chemical inputs required during the major valorization processes. Finally, an optimized pretreatment approach is required for ecological and economically successful textile waste recycling.^110^

### 3.8 Economic Factors

Economic variables are critical in establishing the viability of chemical valorization techniques for textile waste management. The expenses of implementing these procedures, such as energy consumption, chemical use, and required equipment, must be carefully considered. Significant variations in energy usage and operating costs are found when comparing costs across different valorization techniques, and these variations can have a big influence on total profitability. For example, because of the high energy requirements and disposal fees, the cost of traditional waste material burning might range from $100 to $500 per ton.^111^ By reducing the need for fresh catalyst manufacture and the energy input required for reactivation, regeneration-focused catalytic processes, on the other hand, can cut these costs by about 30 to 50%. Additionally, techniques like pyrolysis and anaerobic digestion can turn garbage into useful goods like biofuels or biogas while achieving operating costs of $50 to $300 per ton. When combined, these options not only improve economic efficiency but also support environmental objectives by using less resources and emitting fewer greenhouse gases. Furthermore, market demand for recovered items has a significant impact on overall economic viability, since increased demand leads to higher profitability. The capacity to minimize costs through process optimization, such as efficient solvent recovery or waste minimization, may make these approaches more appealing to industry. Furthermore, the cost of pretreatment and recycling methods varies according to the textile composition and the complexity of the chemicals used.^112−114^ The scalability of these processes also influences economic feasibility, as larger-scale operations can cut per-unit costs, making textile valorization more attractive for widespread adoption. The economic viability of recycling technologies is heavily reliant on growing markets for low-grade textiles, which can be utilized as raw materials to manufacture high-value goods such as natural fiber-reinforced composites. These markets not only assist to value textile waste, but they also help to lessen the environmental impact of landfill disposal. The incorporation of textile waste into composite materials provides a cost-effective alternative, with natural and synthetic fibers serving as reinforcements in high-performance products. However, the difficulty is to develop the most efficient ways for separating and treating these mixed fibers for maximum recovery. Collaborations between industry brands and technology entrepreneurs have been critical to expanding recycling technologies and addressing the growing issue of textile waste.^115^ The complexity and cost of chemical recycling procedures such as solvolysis and depolymerization influence the economic value of synthetic materials such as polyester and nylon. These processes necessitate the use of expensive solvents, catalysts, and large amounts of energy, which raises operational costs. The price volatility of pure synthetic fibers, which is tied to petroleum markets, influences the competitiveness of recycled materials. Contaminants such as dyes and coatings hinder recycling by necessitating costly additional processing. However, technological breakthroughs and increased production capacity can help lower these costs, making synthetic fiber recycling more economically viable.

### 3.9 Additional Factors

Other elements that influence the effectiveness of chemical valorization in textile waste management include reaction time, energy input, and regulatory constraints. Reaction time has a direct impact on overall cost and energy usage, as longer processes can increase operating expenses. Furthermore, energy efficiency is critical, particularly in high-temperature processes such as pyrolysis or gasification, where energy input must be weighed against output to ensure cost-effectiveness. Government restrictions, particularly those governing emissions and waste disposal, have a considerable impact on the methods employed to valorise textile waste. Finally, technical advances in the utilization of catalysts or enzymes can dramatically cut energy and material costs, boosting the recycling process’s sustainability and efficiency.

## 4 Performance Analysis of Chemical Valorization Techniques

### 4.1 Efficiency Metrics

Key performance factors like conversion rates, material recovery, energy efficiency, and overall cost-effectiveness must be taken into account to fully assess the efficiency metrics for chemical valorization procedures in textile recycling. When assessing textile recycling chemical valorization processes, it is essential to take into account critical performance parameters that affect overall efficacy. Conversion rates demonstrate the effectiveness of methods for turning waste textiles into useful products. Indicating the quantity of useable resources extracted, material recovery encourages sustainability and lessens reliance on raw resources. Energy efficiency aims to lower operating expenses and reduce carbon emissions by measuring the energy input in relation to output. Finally, evaluating the market value of recycled goods as well as direct processing expenses is part of overall cost-effectiveness. Researchers and industry participants can improve chemical valorization technologies and promote environmentally friendly textile recycling methods by concentrating on these parameters.^87,116−118^

According to ref (119), cellulose-based textiles can frequently achieve high recovery rates through chemical processes such as acid hydrolysis. According to their research, acid hydrolysis can recover up to 70%+ of cotton fibers. Similar to this, ref (120) stresses that high-purity recovered materials can be obtained from solvent-based techniques, with polyester recovery rates reaching up to 95% when the right solvent mixes and process temperatures are employed. Due to its high recovery rate, which maximizes useable output, it promotes both sustainability and economic objectives. Just as exact curing improves silk’s wrinkle resilience, chemical valorization procedures necessitate meticulously controlled conditions to promote efficiency and preserve material quality.^121^ When evaluating the practicality of chemical recycling methods, energy consumption is a crucial consideration show that enzymatic hydrolysis is significantly more energy-efficient than acid–based techniques in their lifecycle analysis. According to their research, enzymatic techniques use roughly 20% less energy since they function at lower pressures and temperatures, which makes them more appropriate in situations where energy saving is a top priority. It is common practice to assess cost-effectiveness in terms of both operating expenses and the monetary worth of recovered materials. According to ref (122), acid hydrolysis can be cost-effective when used for high-volume textile waste streams because it can handle large quantities of waste efficiently. However, it does have high operational costs because it requires corrosion-resistant equipment and safety measures. In addition to hydrolysis, pyrolysis, and gasification, other chemical processes such as ammonolysis, hydrothermal processing, and glycolysis play an important part in textile waste valorization. These methods enable the effective breakdown of textile waste into valuable products such as monomers, bio-oils, and gases, so promoting sustainable waste management. A comprehensive evaluation of these approaches identifies their uses, benefits, and problems, providing insights into their potential to advance circular economy practices.^123^

### 4.2 Sustainability Evaluation

A thorough analysis of the environmental effects of chemical valorization procedures in textile recycling, including waste reduction, carbon footprint, and energy consumption, is part of the sustainability review process. Recycling procedures must incorporate renewable energy sources in order to improve sustainability and lower the carbon footprint of waste management activities. Recycling facilities can drastically reduce their dependency on fossil fuels by using cleaner energy sources to power their processes, such as solar, wind, or biomass.^124,125^ For instance, biogas produced from organic waste can be used to power machinery or provide heat, and solar panels placed on facility rooftops can offer electricity for sorting and processing equipment. By reducing greenhouse gas emissions linked to conventional energy use, this integration not only reduces operating costs but also enhances the lifespan assessment of recovered materials. In the end, using renewable energy in recycling procedures promotes a circular economy in which resource recovery is accomplished in an ethical and sustainable manner.^126−128^ As environmentally sustainable extraction methods enhance the antibacterial effectiveness of medical bandages, chemical valorization techniques also rely on sustainable procedures to guarantee efficient material recovery.^129^ For sustainable textile waste management, chemical valorization procedures must be calibrated to balance material recovery and energy efficiency, much like how air suction pressure is optimized in compact spinning to produce the highest quality yarn with the least amount of power usage.^130^ This analysis, which draws on a number of studies, is based on four important factors. By dissolving fibers into reusable monomers and components, chemical recycling techniques like depolymerization and acid hydrolysis greatly minimize textile waste. Nearly 90% of processed polyester is transformed into reusable monomers that may be used to create new textile goods, demonstrating the effectiveness of polyester depolymerization in minimizing waste.^120^ This approach supports a circular economy in the textile sector by reducing textile waste and the need for the manufacturing of virgin polyester. According to ref (131), solvent-based extraction reduces waste by recovering a significant portion of the solvents used in the process, even though it is very successful for synthetic fibers. The environmental load is eventually lessened as a result of this recovery since it eliminates the need to dispose of hazardous chemicals and permits additional resource consumption reductions. When evaluating the environmental sustainability of chemical recycling methods, carbon footprint is a crucial indicator. In a lifecycle assessment (LCA) contrasting chemical and enzymatic recycling techniques,^119^ discovered that enzymatic hydrolysis typically has a smaller carbon footprint since it requires less energy. Compared to traditional chemical processes, enzymatic methods utilize less energy and work at lower temperatures, which helps to reduce greenhouse gas emissions.

**Figure 10:**
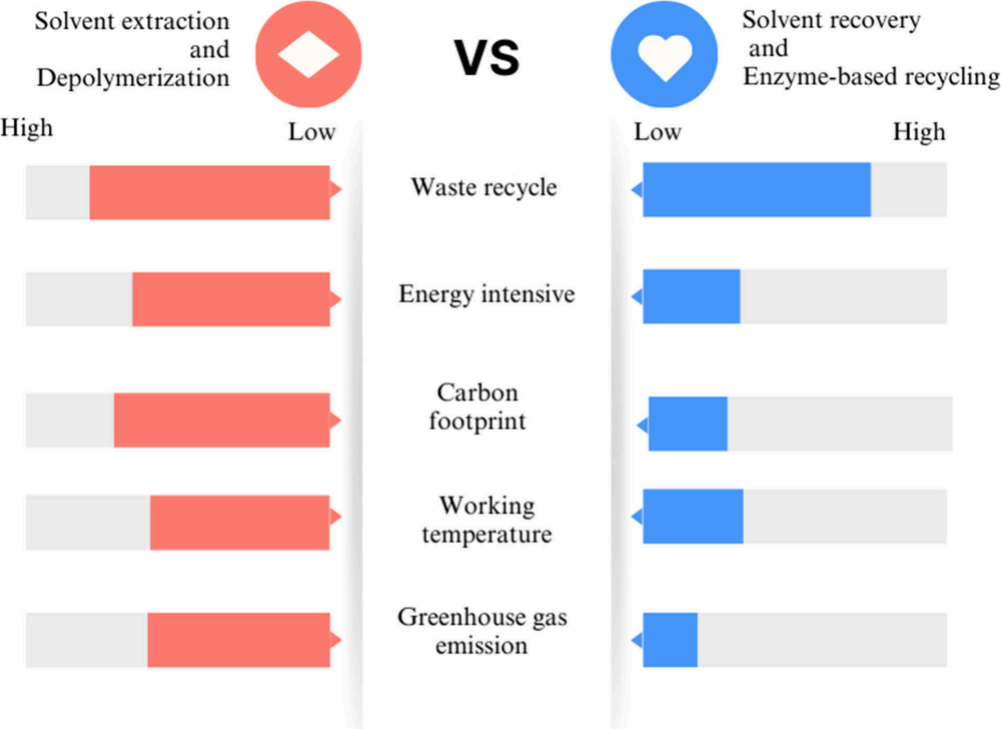
Evaluation of methods on sustainability.

On the other hand, although depolymerization can be energy-intensive, improvements in process efficiency have eventually resulted in lower carbon emissions, according to ref (120). Chemical valorization is a more environmentally friendly method of recycling polyester since it can further reduce carbon emissions by using renewable energy sources to power these recycling operations. One of the main sustainability concerns is energy usage, particularly for energy-intensive processes like solvent extraction and acid hydrolysis. However, by enabling solvent reuse, sophisticated solvent recovery systems can increase energy efficiency and lower the process’s overall energy usage. According to ref (119), because enzymatic recycling processes do not require as much mechanical processing and operate at lower temperatures, they provide a more energy-efficient option than conventional chemical approaches. Because of this distinction, enzymatic techniques for recycling natural fibers are more attractive since they not only require less energy but also have less total environmental impact. In conclusion, evaluation of methods on sustainability a difficult balance between effectiveness and environmental impact is revealed by the sustainability evaluation of chemical valorization procedures (Figure 10). High waste recovery rates can be attained by techniques like solvent extraction and depolymerization, but they may also have high energy and carbon footprint requirements. Nonetheless, advancements in solvent recovery and enzyme-based recycling technologies are opening the door for more environmentally friendly chemical recycling procedures, assisting the textile sector’s transition to circularity.

### 4.3 Comparative Analysis

**Figure 11:**
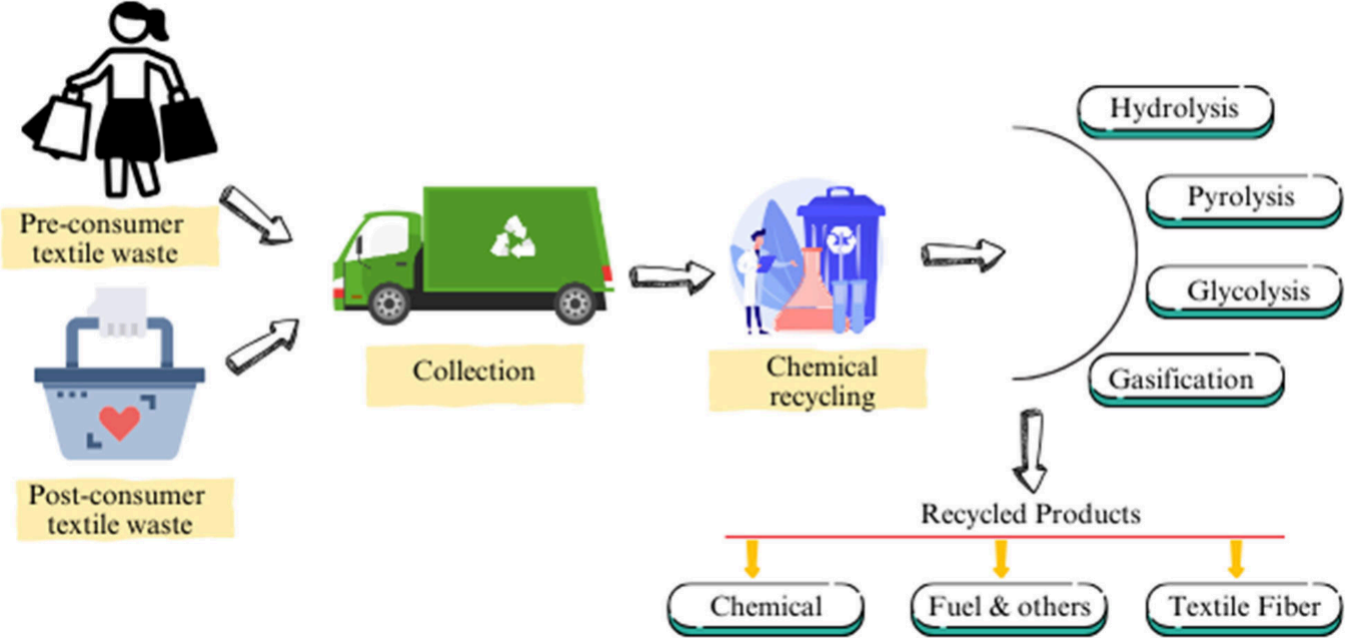
Multistep recycling process.

Effectiveness, scalability, and product quality are important considerations when evaluating the multistep chemical valorization processes of gasification, solvolysis, pyrolysis, and hydrolysis for the recycling of textile waste (Figure 11). For both synthetic (like polyester) and natural (like cotton) fibers, hydrolysis is especially efficient. With high conversion rates, it is excellent in converting polymers into monomers under acidic, alkaline, or enzymatic environments. For recovering textile waste with a defined composition, hydrolysis is therefore perfect.^132,133^

Solvolysis is very successful for synthetic fibers like polyester. Although solvolysis allows for the selective recovery of important components from synthetic textiles, optimal efficiency may necessitate careful control over the solvent type and processing parameters.^134^ Gasification is a method that works well for processing mixed waste streams because it uses a controlled oxygen atmosphere to turn garbage into syngas. The primary outputs are gases and char rather than monomers or polymers that can be utilized to produce new textiles, making it unsuitable for material recovery for textiles.^135^ Although scalable, hydrolysis has drawbacks since it requires particular enzymes or chemical catalysts, which can increase costs and restrict its viability in the industrial setting. However, recent developments are making hydrolysis more suitable for larger-scale processes, including the recycling of polyester.^119^ Because of the advanced technological infrastructure currently in place in the biomass and plastics industries, pyrolysis is one of the most scalable processes. It is ideal for nations with substantial textile waste and centralized recycling systems since it can manage high waste volumes.^132^ Because it relies on the intricacy of solvent handling and recycling systems, solvolysis has moderate scalability. Although it has a bright future for recycling synthetic textiles, its use in mixed or polluted waste streams is limited by its cost and operational complexity.^120^ Gasification can handle enormous amounts of a variety of waste kinds and is very scalable. Although its emphasis on energy generation rather than material recovery makes it less appropriate for textile recycling specifically, it is especially beneficial for centralized facilities managing numerous waste streams.^135^

Renowned for yielding superior monomers with few contaminants, particularly when enzymatic hydrolysis is used. This approach is beneficial for situations where the quality of the final product is crucial since it is perfect for creating materials that can be recycled in the textile sector.^133^ Oils, gases, and char are the products of pyrolysis; these materials have a variety of uses, although they usually need to be further refined for high-purity applications. Since they do not have the material quality needed for textile reprocessing, these byproducts are better suitable for energy production and nontextile applications. Produces high-quality monomers, particularly for polyester, which makes it appropriate for closed-loop recycling systems. However, because contaminants from the textile waste might alter the final product, relying too much on particular solvents may result in differences in product quality.^134^ Syngas, a lower-quality output in terms of material reuse yet helpful for energy production, is the main outcome of gasification. The application of this approach for circular textile recycling is limited because the byproducts are usually not usable in textile manufacture.^135^ Although it has scalability issues, hydrolysis is perfect for recycling single-fiber textiles because it produces high-quality monomers and supports closed-loop recycling. Pyrolysis also can handle a variety of waste types and is scalable, the results are more suitable for fuel than for new textiles. High-quality monomers are produced by solvolysis from synthetic textiles like polyester, but it calls for certain solvents and handling. Although gasification has no direct uses in the manufacturing of textiles, it is very scalable and appropriate for energy recovery from mixed waste streams. In Table 4, the comparison shows that while pyrolysis and gasification are excellent at managing a variety of waste types and are scalable, hydrolysis and solvolysis are better suited to creating high-quality, textile-grade monomers.

**Table 4:** Summary of Factors Affecting Chemical Valorization

Aspect	Technique	Key metrics	Sustainability impact	Strengths	Limitations	Ref.
Conversion rate	Hydrolysis	Up to 90% for polyester	Minimizes waste supports a circular economy	Very efficient for synthetic and natural fibers	Requires specific catalysts, potentially high costs	(136)
	Solvolysis	Polyester recovery rates up to 95%	Reduces the need for virgin materials	Selective recovery, high purity	Needs careful control over solvents	(137)
	Acid hydrolysis	Up to 70% for cellulose-based textiles	Promotes sustainability through high recovery rates	Efficient for high-volume textile waste	High operational costs due to equipment requirements	(138)
	Gasification	Produces syngas, not suitable for material recovery	Focuses on energy recovery, less suitable for textiles	Scalable for mixed waste streams	Low quality of byproducts for textiles	(139)
	Pyrolysis	Produces oils and gases	Primarily for energy production, limited textile recovery	Scalable, handles various waste types	Not suitable for textile-grade applications	(140)
Energy efficiency	Enzymatic hydrolysis	20% less energy than acid-based methods	Reduces carbon footprint through lower energy demands	Attractive for natural fibers	Slower process than conventional methods	(141)
	Acid hydrolysis	Higher energy costs for operation	High energy use, but can handle large volumes	Cost-effective for high-volume streams	Equipment must be corrosion-resistant	(142)
Material recovery	Chemical process	High purity of recovered materials	Improves resource efficiency	Hydrolysis and solvolysis yield high-quality monomers	Potential contamination from mixed waste	(143)
	Enzymatic processes	Efficient breakdown, minimal contamination	Lower environmental impact	Superior product quality for recycling	Limited to specific types of fibers	(144)
Carbon footprint	Enzymatic hydrolysis	Lower emissions due to reduced energy use	Helps in reducing greenhouse	N/A	N/A	(145)

## 5 Current Challenges

**Figure 12:**
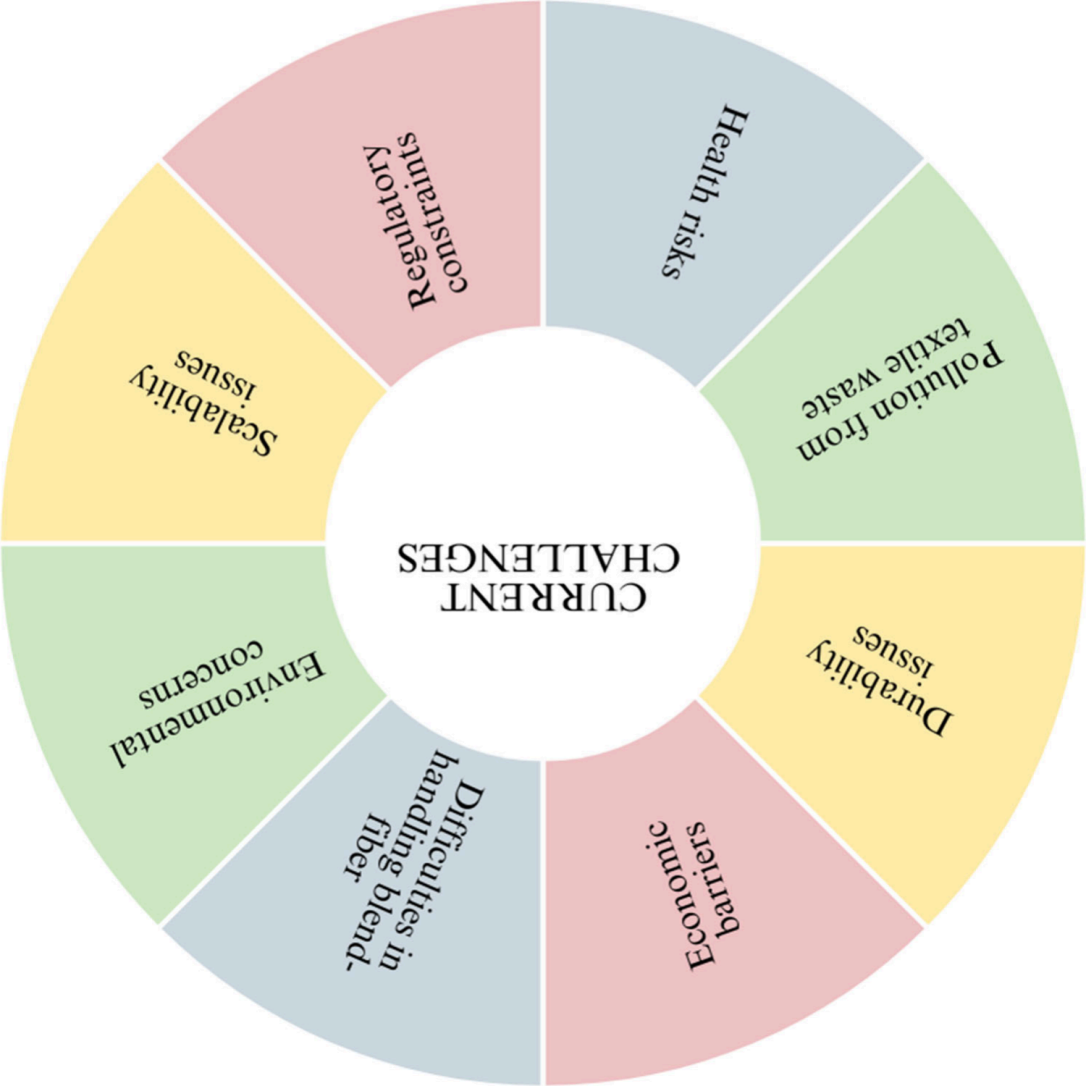
Challenges in textile waste recycling.

The recycling of textiles is a multistep, intricate process that includes gathering, sorting, processing, and production. Because each step involves a new set of tools and knowledge, creating a simplified and effective process creates many challenges (Figure 12). For instance, one of the biggest recyclers of textile waste worldwide is Trans-America Trading Company, which has its headquarters in the United States, with the ability to handle more than 5.5 million kg of textile waste annually. Sorting and classifying these massive amounts of, typically mixed, textile waste into over 300 different groups according to size, content, and fiber type is a major task. To finish this procedure on a wide scale, a great deal of labor and time are needed.^146^

### 5.1 Difficulties in Handling Blend-Fiber Textiles

Particularly for blended fibers, the distinctive characteristics of these materials present significant obstacles to the valorization of textile waste. Blended fibers, which can be any combination of natural and synthetic components, cotton–polyester, wool–polyester, nylon cotton, or any other combination, have different chemical properties and recycling requirements.^147^ Like, cotton and silk, two venerable natural fibers, bring their unique characteristics to textile blends.^148^ Similar to how temperature and mordanting affect the silk–cotton blend’s ability to dye, maximizing process parameters in chemical valorization procedures is essential to overcoming blended fiber composition issues and achieving effective recycling. The primary reason for these challenges is the diversity of chemical structures^131^ present in the fibers used in these blends, which necessitates the employment of advanced recycling techniques to effectively separate and manage each fiber component.^149^ Materials with different chemical properties, such as synthetic polymers like polyester and nylon, and natural fibers like cotton and wool, combine to form blend fibers. Because synthetic fibers are made of polymers generated from petrochemicals,^150^ whereas natural fibers are cellulose- or protein-based,^151^ their recycling processes are essentially different. For instance, polyester needs glycolysis or methanolysis to depolymerize it into monomers like terephthalic acid and ethylene glycol, whereas cotton can be chemically recycled using enzymatic hydrolysis or alkaline treatments that break down cellulose into glucose.^147^ Because neither of these processes can be recycled simultaneously without sacrificing fiber quality, there is a bottleneck caused by their incompatibility. Being able to distinguish between different components is essential for the effective recycling of blended fibers. However, because of the close intertwining of fibers, physical separation alone is not always possible. For instance, wool (a protein-based fiber) mixed with polyester resists chemically the same treatments as polyester does.^150,152^ In order to dissolve the polyester without harming the wool, the separation process frequently calls for the use of certain solvents or alkaline hydrolysis, which is an expensive and time-consuming procedure.^153^

Due to the lack of selectivity in many chemical recycling procedures for mixed fibers, one kind of fiber may partially degrade while the other remains intact. For instance, in blends of nylon and cotton, enzymes can effectively hydrolyze the cotton while essentially leaving the nylon intact. To preserve the integrity of the individual fibers during recycling, depolymerization processes need to be precisely calibrated.^154^ Blends that include elastane, which is frequently added to fabrics to give them stretch qualities, present an additional challenge. Because of its extreme tenacity and chemical resistance, elastane is difficult to recycle or break down using standard methods. It is frequently necessary to use solvent-based methods in order to dissolve elastane while protecting other fibers, such as cotton.^155^ However, the cost and environmental consequences of these solvents further complicate the recycling process.^156^ Furthermore, the remaining chemicals from the separation process may degrade the recycled fibers’ quality and make it less profitable for them to be used again in premium fabrics. The high costs of chemical treatments and fiber separation make the value-adding of blended textile waste not only technically difficult but frequently economically unfeasible. These activities may also result in secondary waste streams that require management, which could have negative effects on the environment. As a result, blend fibers pose a substantial challenge because of their composite nature, even though the valorization of pure fiber textiles is comparatively better progressed.^157^

### 5.2 Environmental Issues with the Valorization of Textile Waste

The goal of textile waste valorization is to turn trash into profitable goods, however, the complicated and sometimes dangerous recycling procedures involved pose serious environmental challenges. These problems are made more complicated by the growing use of synthetic materials and blended fibers in textiles, which raises environmental concerns about waste production, chemical pollution, and energy use.^157^ One of the main ways that blended textile fibers, such as cotton–polyester or nylon–wool, are recycled, is by chemical procedures that pollute the environment. Toxic solvents or chemicals that can release hazardous byproducts into the environment are used in many recycling processes. For instance, volatile organic compounds (VOCs), which contribute to air pollution and have negative impacts on human health and local ecosystems, are frequently used in solvent-based extraction procedures used to dissolve polyester in cotton–polyester blends.^147^ Similarly, improper management of the chemical waste produced by alkaline hydrolysis, a popular method for recycling nylon blends, can result in water pollution.^154^

Blended textile recycling also produces a lot of waste, which is difficult to get rid of in an eco-friendly way. Depolymerization is one of the chemical separation techniques that frequently results in secondary waste streams with residues that need to be treated further. This enhances the potential for soil and water pollution if these waste streams are not appropriately handled, in addition to making recycling more expensive and complicated. Additional environmental difficulties arise, for example, when wool–polyester blends treated by alkaline hydrolysis leave behind hazardous residues that need to be neutralized before disposal.^157^ Blended textile fiber recycling frequently requires energy-intensive procedures that primarily rely on heat, pressure, or chemical reactions. The ecology is further impacted by this high energy use, which increases the carbon footprint. Reference (158) points out that the recycling of polyester, for example, usually entails high-temperature glycolysis or methanolysis processes, which require significant energy input and raise greenhouse gas emissions. Further aggravating the environmental impact are recycling procedures for blends including elastane, which call for specialized solvents that are expensive to extract and challenging to recycle. A large number of the chemicals used in recycling procedures are difficult to recover and reuse because of the complicated composition of blended fabrics. Solvents used to extract elastane from cotton in situations like cotton–elastane blends are frequently nonrecoverable,^156^ increasing chemical waste and contaminating the environment. Because these solvents must be continuously supplied, which further impacts the environment, the inability to effectively recycle these chemicals not only increases the waste load but also reduces the economic viability of textile waste valorization.^122^

### 5.3 Economic Obstacles to the Valorization of Chemical Textile Waste

The practicality of chemical textile recycling is limited, particularly for blended fibers, due to major cost obstacles. High input costs, significant energy demands, the requirement for infrastructure, and difficulties with chemical waste disposal are the main causes of these obstacles.^157^ The process is made economically difficult and, in many circumstances, unprofitable by these issues together, which prevents textile waste valorization from being widely adopted and from scaling up.^160^ Blended fiber chemical recycling frequently requires expensive, specialized reagents or solvents. For instance, certain solvents are needed to dissolve polyester in procedures that extract it from cotton–polyester blends;^147^ these solvents are expensive and difficult to recycle. Additionally, these solvents may deteriorate with usage, necessitating new supplies for every batch, hence raising the total cost claim that these solvent systems are too costly for widespread use since they also provide logistical challenges for handling, storing, and disposing of the solvents.^161^

Synthetic fiber chemical recycling processes like nylon depolymerization and polyester glycolysis need a lot of energy and high temperatures. By raising greenhouse gas emissions, this energy demand drives up operating expenses and negates the environmental advantages of recycling. Reference (150) highlights that these energy-intensive methods, particularly for polyester blends, result in significant fuel and electricity expenses, which reduces the process’s viability from an economic standpoint. According to Mishra, cutting the energy required for recycling operations is crucial, but the efficiency of present technologies is insufficient to significantly lower prices It costs a lot of money to set up chemical recycling facilities since they need specialized machinery, space, and technology. Methods such as alkaline hydrolysis and methanolysis require specialized equipment and regulated conditions to handle various fibers; these are expensive to set up and keep running.^162^ Furthermore, many current facilities are ill-suited to manage the particular requirements of textile recycling, as ref (122) explains. This forces operators to make costly investments in new technologies. For example, elastane recycling facilities need to have certain solvent recovery systems, which raises infrastructure costs and makes these operations difficult financially.^160^ Prior to disposal, additional treatment is necessary to neutralize hazardous byproducts when handling leftover chemicals from techniques such as alkaline hydrolysis employed on wool–polyester blends.^152^ A study^161^ found that trash treatment, particularly in areas with strict environmental legislation, can account for a significant amount of the total recycling cost. Recycled fibers may experience quality degradation, which reduces their appeal for performance- or high-end-oriented applications and affects profitability. Another two studies^157,162^ show that, particularly when dealing with chemically processed fibers that might not meet the performance standards needed for commercial applications, the market price for recycled fibers is frequently insufficient to cover the recycling costs. Furthermore, recycled products face difficulties in gaining market traction due to competition from low-cost virgin fibers, especially polyester, which further reduces the financial returns on recycling efforts.

### 5.4 Difficulties with Chemical Methods for Valorizing Textile Waste

**Figure 13:**
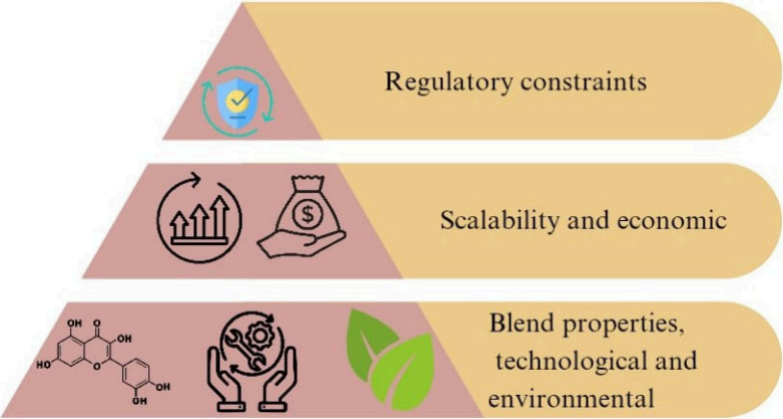
Pyramid structure of certain challenges.

The difficulties include scaling concerns, legislative restrictions, customer perceptions, environmental risks, and technology limitations and the range of challenges follows a pyramid structure (Figure 13): Pyramid Structure of Certain Challenges Effectiveness. It is imperative to tackle these obstacles in order to progress chemical recycling’s efficacy and uptake in textile waste management.^160^ Since many of the chemical recycling techniques used today are material-specific, they might not be suitable for the vast range of fibers found in textile waste streams. Examples of processes that work well for polyester are glycolysis and methanolysis, however, they do not work well for natural fibers like cotton or wool. These material variances make it challenging to develop a universal chemical recycling solution, and new technologies are required to handle the specific qualities of various textile fibers.^164^

Solvents, catalysts, and other hazardous, combustible, or caustic compounds are used in a lot of operations. Workers in recycling plants may be at risk from these compounds if appropriate safety precautions are not taken. Insufficient containment may also lead to chemical spills, which could be detrimental to the ecosystems around. For instance, volatile organic compounds (VOCs) from solvents used in polyester recycling can pollute the air, affecting the health of neighboring residents as well as the quality of the local air.^165^ It is challenging to use the technologies on an industrial scale due to the associated costs and operational complexity. For instance, the cost of producing and optimizing enzymes makes enzymatic hydrolysis—which has shown promise for cotton recycling—unaffordable for large-scale applications.^166^ The need for specialized infrastructure and equipment, which raises both capital and operating expenses, further impedes the scalability of these procedures.^164^ Innovative solutions to the scalability issues frequently encountered in sophisticated recycling and waste management systems are provided by modular reactor designs and remote processing facilities. Because of their smaller, transportable units, modular reactors can be installed in different places to meet local needs, which lowers transportation costs and emissions related to waste transit. These reactors allow for gradual expansion, meaning that more modules can be added as processing capacity demands increase without requiring a major overhaul of the current infrastructure.^167,168^ Additionally, by offering localized solutions that improve resource recovery while reducing environmental consequences, decentralized processing facilities can strengthen communities. These facilities encourage local recycling programs, which not only lessen the carbon footprint of long-distance waste transportation but also increase community involvement and understanding of sustainable practices.^169,170^ When combined, these strategies enable a recycling ecosystem that is more responsive and flexible, able to successfully satisfy a range of regional demands and aid in the shift to a circular economy.^171^ Regulations pertaining to the disposal of hazardous byproducts, for instance, may subject recycling plants to increased expenses and operational restrictions. Textile waste valorization is further complicated in certain areas by stringent environmental rules that restrict the kinds of chemicals that can be used or incur fines for noncompliance.^172^ International textile recycling operations might be complicated by the fact that these regulatory hurdles can differ greatly between nations.^122^ Numerous fabrics are treated with waterproofing chemicals, flame retardants, and dyes, which can obstruct the recycling process.^163^ For example, dye residues may react with recycling chemicals, lowering the recovered material’s quality and restricting its uses. The procedure of decontaminating these fabrics is made more complex and expensive by the need for extra chemical or mechanical stages.^162^ Chemically recycled fibers may perform and last less well than virgin fibers, which would limit their use.^173^ The chemical procedures employed in recycling may cause this deterioration by dissolving fiber structures or eliminating crucial fiber characteristics. Recycled polyester, for instance, could be less elastic and strong, which makes it less appropriate for some high-performance uses. Due to this difficulty, it is challenging to compete with virgin fibers, especially in sectors that value durability, including technical textiles and outdoor gear.^163^ Other challenges with chemical methods for valorizing textile waste include ensuring consistent deposition and treatment effectiveness, as demonstrated by the need for multiple layers or concentrated applications of herbal-synthesized colloidal silver nanoparticles to achieve optimal UV protection and fiber surface coverage^174^ (merge with reference no. (102)). Table 5 shows a summary on current challenges in valorizing textile waste. In the same way as improving nanoparticle treatments improves UV protection on cotton fabric, chemical valorization procedures must handle complicated materials and creative processes to optimize recycling effectiveness and sustainability.

**Table 5:** Summary of Current Challenges

Challenge area	Key points	Ref.
Difficulties in handling blend-fiber textiles	Complex composition of blended fibers (e.g., cotton–polyester, wool–polyester)	(153)
	Different chemical properties require advanced recycling techniques	(158)
	Incompatibility in recycling processes leads to bottlenecks	(160)
	Physical separation is often inadequate, necessitating costly chemical methods	(175)
	Toxic solvents and chemicals contribute to air and water pollution	
	Secondary waste streams from chemical recycling pose contamination risks	
Environmental issues with valorization	Energy-intensive processes increase carbon footprint and greenhouse gas emissions	(105, 111, 176, 177)
	Difficulty in recovering and reusing many solvents increases waste	
	High input costs for specialized chemicals and energy demands hinder feasibility	
Economic obstacles to valorization	Significant infrastructure costs for establishing recycling facilities	(125, 178, 179)
	Competition from low-cost virgin fibers reduces the market viability of recycled fibers	
	Current recycling methods are often material-specific, limiting applicability	
Additional difficulties with chemical methods	Hazardous chemicals increase safety risks for workers	(180)
	Regulatory compliance adds operational complexity and costs	(179)
	Treated fabrics with additional chemicals complicate the recycling process	(137)

## 6 Future Opportunities

The growing population and rising consumption rates result in increased volumes of textile waste, which exacerbates pollution levels. The diversity of textile finishes and colors poses issues for recycling procedures since specific treatments are necessary to appropriately control these variances. Cotton is easily recycled using acid hydrolysis, whereas polyester is better suited to base hydrolysis. Enzyme treatments offer a long-term solution for decreasing pollutants from chemicals and dyes used in textile production. Pyrolysis is an alternate approach that utilizes thermal breakdown to transform textile waste into bio-oil, gases, and char. Solvent-based recycling is required for fiber mixes, however, a suitable solvent must be used to retain fiber quality. While enzymatic procedures are environmentally friendly, they are sometimes constrained by expensive prices and sluggish reaction times. Chemical hazards, regulatory difficulties, and high operational costs all pose challenges for large-scale recycling. The presence of elastane and other mixed fibers complicates recycling operations and increases their cost. Moving toward sustainable waste management will require the development of scalable technologies, effective pretreatment methods, and optimized catalysts. Modern techniques for recovering sugars as monosaccharides from natural fiber waste, especially cellulosic materials, are being used to transform textile waste into biobased goods.^181^ The circular economy’s approach to textile waste valorization offers the chance to turn waste materials into useful products, protecting resources, lessening environmental effect, encouraging sustainable business practices, and changing fashion industry production and consumption patterns for a more environmentally conscious and regenerative future.^182^ By using less water and energy than conventional chemical methods, the application of enzymatic processing in textile waste valorization presents significant opportunities, positioning biotechnologies as a sustainable alternative for emerging markets to enhance economic and environmental outcomes. Strategic management frameworks can assist in overcoming resistance to adopting these technologies, ensuring a more sustainable future for the textile industry.^183^ By developing novel technologies that can handle a wide range of textile types and blends, the valorization of textile waste offers opportunities for significant advancements in chemical processes. These techniques have the potential to reduce landfill waste and produce value-added products, but their success hinges on the application of life cycle assessments to assess the environmental, social, and economic impacts and make sure that the selected pathways support the local circular economy.^184^ The treatment of hydrothermal liquefaction wastewater has led to recent advances in chemical waste valorization, including the use of cutting-edge techniques like anaerobic digestion, microbial fuel cells, microbial electrolysis cells, and supercritical water gasification. These techniques, when combined, offer promising pathways for the recovery of valuable bioenergy sources, nutrient extraction, and the reduction of environmental pollutants. This emphasizes the significance of choosing the right technologies based on the biomass’s characteristics.^185^ A number of recent developments in chemical waste valorization of textile waste through anaerobic digestion have been made possible by the separation of polyester from polycotton, the use of solvent and advanced oxidation processes for improved methane yield, and the application of chemical, biological, and thermal pretreatments. These advances also optimize the production of biogas from textile waste. It is recommended that future study concentrate on creating effective pretreatment processes, investigating novel codigestion approaches, improving methods for removing dye from textiles, and including life cycle assessments in order to analyze the total environmental and financial effects of textile waste extraction.^186^

Thermodynamics and reaction kinetics are essential for recycling process optimization in textile waste valorization. First-order reaction models, in which the rate constant (*k*) fluctuates with temperature in accordance with the Arrhenius equation (*k* = *A*e^–*E*_a_/*RT*^), are frequently used to characterize the kinetics of hydrolysis and depolymerization of cellulose-based fibers. Because more molecules collide at higher temperatures, reaction rates are accelerated. Thermodynamically, Gibbs free energy (Δ*G*) evaluations aid in the identification of spontaneous processes; under certain circumstances, a negative (Δ*G*) indicates feasibility. For procedures like pyrolysis or supercritical fluid extraction that attempt to transform polymers into more useful monomers or oligomers, enthalpy (Δ*H*) and entropy (Δ*S*) changes must be evaluated. Researchers can enhance material recovery from textile waste and advance sustainable textile management methods by incorporating kinetic and thermodynamic insights into process design.^187,188^

The utilization of biotechnological techniques to convert textile waste, specifically cotton and cotton–polyester blends, into valuable secondary raw materials and platform chemicals holds the key to the potential for expanding chemical valorization processes and incorporating them into the textile and waste management industries. This will be accomplished while maintaining the techniques’ economic viability and environmental sustainability.^189^ A substantial amount of waste is generated during the production process, including yarn leftovers primarily from synthetic fibers and mixed material fabric waste, which could potentially be turned into new products if barriers like the lack of a market and adequate recycling technology for mixed materials are addressed. The textile industry presents significant opportunities for implementation by recognizing postconsumer waste as a valuable resource for producing new textile products.^190^ While academic institutes contribute state-of-the-art scientific information and technical developments, industries offer practical insights into market needs, scaling issues, and operational efficiencies.^191^ Developing more effective recycling procedures, improving catalyst performance, or producing novel materials that improve recyclability are some examples of collaborative efforts.^192^ Joint efforts can also make it easier to obtain funding for pilot projects and demonstrations that confirm these technologies’ efficacy in practical contexts. Through workshops and training, these collaborations also foster knowledge transfer, guaranteeing that workforce competencies meet new technology demands. Stakeholders can build a strong ecosystem that expedites the shift to sustainable chemical recycling solutions while tackling urgent environmental issues related to waste management by encouraging close cooperation between academia and industry.^191,193^ The development of efficient pretreatment techniques that improve enzymatic saccharification and enable the conversion of different cotton-based and blended cotton–PET textile wastes into high-yield value-added chemicals like bioethanol, sorbitol, lactic acid, terephthalic acid, and ethylene glycol illustrates the importance of integrating chemical valorization processes into the textile and waste management industries. This approach also highlights the efficacy of an integrated biorefinery approach for sustainable textile waste valorization.^194^ The significance of incorporating chemical valorization processes into the textile and waste management industries is demonstrated by the development of effective pretreatment techniques that enhance enzymatic saccharification and permit the conversion of various cotton-based and blended cotton–PET textile wastes into high-yield value-added chemicals like bioethanol, sorbitol, lactic acid, terephthalic acid, and ethylene glycol. This strategy also demonstrates the effectiveness of an integrated biorefinery strategy for the valuable extraction of sustainable textile waste.^110^ Supporting improvements in chemical valorization requires strong regulatory and policy frameworks. Through tax credits, grants, and subsidies for businesses investing in recycling technologies, governments can encourage innovation.^123,195^ A circular economy can be supported by explicit regulations that place a higher priority on chemical valuation than on conventional trash disposal. Simplifying safety evaluations for novel materials produced through valorization procedures will promote quick adherence to environmental regulations. Furthermore, enforcing extended producer responsibility (EPR) laws will encourage the creation of recyclable materials by holding producers responsible for the full lifecycle of their goods.^123,195^ In order to promote public–private partnerships for research projects centered on sustainable feedstocks and catalytic processes, as well as to unify rules and ease international trade in recycled materials, international cooperation is essential. Together, these initiatives can support sustainability objectives and foster an innovative atmosphere.^176,179^

## 7 Conclusion

There is a lot of potential for improving sustainability and fostering a circular economy in the textile sector through the value-adding of textile waste. Although the diverse makeup of textile waste—which includes chemical additives and blended fibers presents significant obstacles, new chemical and enzymatic recycling methods provide promising avenues for efficient resource recovery. Important techniques including pyrolysis, hydrolysis, and enzymatic treatments show promise for turning textile waste into products with additional value, especially when complemented by sophisticated solvent recovery systems, focused pretreatment plans, and optimized catalysts. Scalability and economic viability, however, continue to be major challenges that call for more development and improvement. Reducing the impact on the environment, saving resources, and improving economic sustainability in textile waste management all depend on addressing these issues. These value-adding procedures’ ongoing advancement and integration are expected to contribute substantially to global environmental sustainability.

## Glossary and Abbreviations

BOD (Biochemical Oxygen Demand): A measure of the amount of oxygen required by microorganisms to decompose organic matter in water
COD (Chemical Oxygen Demand): The total amount of oxygen required to oxidize both organic and inorganic substances in water
TPA (Terephthalic Acid): A chemical compound used as a raw material in the production of polyester
PET (Polyethylene Terephthalate): A common type of polyester used in textiles and packaging
Pyrolysis: A thermal decomposition process conducted in the absence of oxygen to convert organic material into gases, bio-oils, and char
Gasification: A thermochemical process that converts carbon-based materials into syngas by exposing them to high temperatures in an oxygen-limited environment
Enzymatic hydrolysis: The process of breaking down polymers into monomers using specific enzymes, often aplied to cellulose-based textiles
Glycolysis: A chemical process used to depolymerize polyester into its monomers, terephthalic acid, and ethylene glycol
Syngas: A mixture of hydrogen, carbon monoxide, and other gases produced through gasification, often used for energy generation
BTXS (Benzene, Toluene, Xylene, Styrene): A group of aromatic hydrocarbons often analyzed in pyrolysis products
LCA (Life Cycle Assessment): A methodology for assessing the environmental impacts associated with the stages of a product’s life
VOC (Volatile Organic Compound): Organic chemicals that easily evaporate at room temperature and contribute to air pollution
